# Lateral Orbitofrontal Cortex and Basolateral Amygdala Regulate Sensitivity to Delayed Punishment during Decision-Making

**DOI:** 10.1523/ENEURO.0170-22.2022

**Published:** 2022-09-06

**Authors:** Anna E. Liley, Daniel B. K. Gabriel, Nicholas W. Simon

**Affiliations:** Department of Psychology, University of Memphis, Memphis, TN 38152

**Keywords:** basolateral amygdala, decision-making, delay discounting, lateral orbitofrontal cortex, punishment

## Abstract

In real-world decision-making scenarios, negative consequences do not always occur immediately after a choice. This delay between action and outcome drives the underestimation, or “delay discounting,” of punishment. While the neural substrates underlying sensitivity to immediate punishment have been well-studied, there has been minimal investigation of delayed consequences. Here, we assessed the role of lateral orbitofrontal cortex (LOFC) and basolateral amygdala (BLA), two regions implicated in cost/benefit decision-making, in sensitivity to delayed versus immediate punishment. The delayed punishment decision-making task (DPDT) was used to measure delay discounting of punishment in rodents. During DPDT, rats choose between a small, single-pellet reward and a large, three-pellet reward accompanied by a mild foot shock. As the task progresses, the shock is preceded by a delay that systematically increases or decreases throughout the session. We observed that rats avoid choices associated with immediate punishment, then shift preference toward these options when punishment is delayed. LOFC inactivation did not influence choice of rewards with immediate punishment, but decreased choice of delayed punishment. We also observed that BLA inactivation reduced choice of delayed punishment for ascending but not descending delays. Inactivation of either brain region produced comparable effects on decision-making in males and females, but there were sex differences observed in omissions and latency to make a choice. In summary, both LOFC and BLA contribute to the delay discounting of punishment and may serve as promising therapeutic targets to improve sensitivity to delayed punishment during decision-making.

## Significance Statement

Negative consequences occurring after a delay are often underestimated, which can lead to maladaptive decision-making. While sensitivity to immediate punishment during reward-seeking has been well-studied, the neural substrates underlying sensitivity to delayed punishment remain unclear. Here, we used the delayed punishment decision-making task (DPDT) to determine that lateral orbitofrontal cortex (LOFC) and basolateral amygdala (BLA) both regulate the discounting of delayed punishment, suggesting that these regions may be potential targets to improve decision-making in psychopathology.

## Introduction

Many psychiatric diseases are characterized by insensitivity to detrimental outcomes (Bechara, 2005; Hartley and Phelps, 2012; Jean-Richard-dit-Bressel et al., 2019; Orsini and Simon, 2020). One factor that drives this insensitivity is the presence of a delay that often precedes occurrence of these outcomes (Murphy et al., 2001; Bechara et al., 2002; Field et al., 2019). For example, individuals with substance misuse problems seek out drugs to receive immediate positive reinforcement, but often underestimate impending withdrawal symptoms or financial/legal concerns that occur later in time (Bickel et al., 2014a; Dalley and Ersche, 2019). This can be attributed to “delay discounting,” wherein the motivational value of delayed outcomes is underestimated compared with immediate outcomes.

While delay discounting of rewards has been well-studied (Floresco et al., 2008; Kable and Glimcher, 2010; Mar et al., 2011; Bickel et al., 2014b; Burton et al., 2014; Frost and McNaughton, 2017), there is minimal research investigating the neurobiological mechanisms underlying sensitivity to immediate versus delayed punishment. Furthermore, preclinical research on punished reward-seeking has primarily focused on consequences that occur immediately after an action (Pollard and Howard, 1979; Jonkman et al., 2012; Orsini et al., 2015a; Park and Moghaddam, 2017; Jean-Richard-Dit-Bressel et al., 2018; Rodríguez et al., 2018; Halladay et al., 2020). To address this discrepancy, we developed the rat delayed punishment decision-making task (DPDT), which offers choice between a small reward and a large reward followed by a mild foot shock. The shock initially occurs immediately after choice, but is preceded by a systematically escalating delay as the task progresses (Liley et al., 2019). Rats initially avoid the punished option, then shift preference toward the punished option as delays increase, thereby demonstrating delay discounting of the negative motivational value as a function of delay. Moreover, despite comparable decision-making with immediate punishment, males discount delayed punishment to a greater degree than females. Additionally, delay discounting of punishment is not correlated with discounting of rewards, suggesting that these processes may be governed by divergent neurobiological mechanisms (Liley et al., 2019).

The lateral orbitofrontal cortex (LOFC) and basolateral amygdala (BLA), two brain regions with dense reciprocal connections (Price, 2007), are two candidates that likely drive discounting of delayed punishment during reward-seeking. OFC is a prefrontal cortical brain region that receives input from all major sensory systems in addition to influences from limbic regions (Mcdonald, 1991; Carmichael and Price, 1995), making it an optimal site for integration of perceptual and emotional information to guide decision-making. LOFC is involved with a number of cognitive processes that are important for cost-benefit decision-making (Padoa-Schioppa and Assad, 2006; Karimi et al., 2019), including reward/punishment integration (Morrison, et al., 2011; Orsini et al., 2015b; Jean-Richard-Dit-Bressel and McNally, 2016). Additionally, LOFC is involved with sensitivity to delayed rewards (Mobini et al., 2002; Roesch et al., 2006; Zeeb et al., 2010); despite the lack of correlation between delayed reward and punishment discounting, it is feasible to speculate that brain regions that encode delays in rewarding contexts contribute to delay processing with punishment, even if processing differs based on rewarding/aversive valence. BLA regulates punishment-induced suppression of reward seeking and is involved with attribution of salience to aversive cues (Jean-Richard-dit-Bressel and McNally, 2015; Piantadosi et al., 2017; Hernandez et al., 2019). BLA is also involved with goal-directed behavior and encodes outcome-specific values to guide actions (Wassum and Izquierdo, 2015). Additionally, it associates cues with rewards during events, making this region imperative for using past experiences to flexibly guide decisions for optimal outcomes (Schoenbaum et al., 2000). Thus, both regions likely contribute to integrating delays with punishment to guide decision-making.

Here, we separately assessed the involvement of LOFC and BLA in sensitivity to delayed punishment during decision-making using pharmacological inactivation of each region before DPDT testing. We also compared the effects of LOFC and BLA inactivation between male and female rats. Finally, to test whether effects of inactivation were influenced by task design or impaired behavioral flexibility, LOFC and BLA were individually inactivated before a modified version of DPDT with descending punishment delays (REVDPDT).

## Materials and Methods

### Subjects

A total of 73 Long–Evans rats obtained from Envigo were aged 70 d on arrival and used for these experiments (total LOFC: *n* = 38, female: 18, male: 20; total BLA: *n* = 35, female: 15, male: 20). Rats were restricted to 85% free feeding weight one week before behavioral training to encourage motivation and pursuit of rewards during task performance. Free-feeding weights were altered throughout the experiment in accordance with Envigo growth charts to account for growth. All rats were individually housed and maintained on a 12/12 h reverse light/dark cycle. All methods were approved by the University of Memphis Institutional Animal Care and Use Committee.

### Surgery

Before behavioral assessments, rats underwent bilateral cannulation surgery for either LOFC (+3.0 mm AP, +3.2 ML, and −5.5 DV from skull surface; edited from Roesch et al., 2006; Paxinos and Watson, 2013) or BLA (−3.0 mm AP, +5.0 ML, and −8.7 DV from skull surface; edited from Orsini et al., 2015b; Paxinos and Watson, 2013) infusions. Rats were anesthetized in an isoflurane gas induction chamber, then placed into a stereotaxic apparatus (Kopf) while resting on a heating pad adjusted to 40°C. Isoflurane was provided throughout surgery via a nose cone. Cannulae were held in place by a dental cement headcap anchored by three bone screws. Upon completion of surgery, rats were subcutaneously given 1 ml of sterile saline, and a solution of acetaminophen and H_2_O in a dish to moisten food during recovery. Rats were closely monitored for signs of infection or distress during the next week, with cage bedding changed daily for the first 3 d.

### Behavior apparatus

Testing was conducted in standard rat behavioral test chambers (Med Associates) housed within sound attenuating cubicles. Each chamber was equipped with a recessed food pellet delivery trough fitted with a photograph beam to detect head entries, and a 1.12-W lamp to illuminate the food trough. Food pellets were delivered into the food trough 2 cm above the floor centered in the side wall. Two retractable levers were located on the left and right side of the food trough 11 cm above the floor. A 1.12-W house light was mounted on the opposing side wall of the chamber. Beneath the house light was a circular nose poke port equipped with a light and photograph beam to detect entry. The floor of the test chamber was composed of steel rods connected to a shock generator that delivered scrambled foot shocks. Locomotor activity was assessed throughout each session with infrared activity monitors located on either side of the chamber just above the floor. Test chambers are interfaced with a computer running MedPC software, which controlled all external cues and behavioral events.

### Shaping procedures

Food restriction and behavioral training began after one week of recovery. Before acquisition of DPDT, rats underwent a series of shaping procedures. Rats were first taught to associate the food trough with food pellets during magazine training. In separate sessions, rats then trained to press a single lever (left or right, counterbalanced across groups) to receive one pellet of food. After performing 50 reinforced lever presses within 30 min, rats trained to press the opposite lever under the same criterion. Following this were shaping trials in which both left and right levers were retracted, and rats were required to nose poke into the food trough during a period of illumination from both the house and food trough lights. Nose poking evoked the extension of a single lever (either left or right in pseudorandom order). Each subsequent lever press was reinforced with a single pellet, along with extinguishing of house and trough lights and retraction of the lever. After achieving a minimum of 30 presses of each lever in a 60-min time span, rats progressed to magnitude discrimination training.

The 30-min reward magnitude sessions used two levers with counterbalanced presses producing either one or three pellets. As in the previous stage of training, each trial began with the illuminated house and trough lights, after which a nose poke into the trough led to extension of one or both levers. A press on one lever produced a single pellet while the other produced three, followed by lever retraction, termination of all cues, and progression to a 10 ± 2-s intertrial interval (ITI). There were five blocks of 18 trials in each task, with the first eight providing only a single lever (forced choice) the next 10 both levers (free choice). Once rats achieved >75% preference for the large reward during free choice trials, they began either DPDT or REVDPDT training.

### DPDT

**Figure 1.**
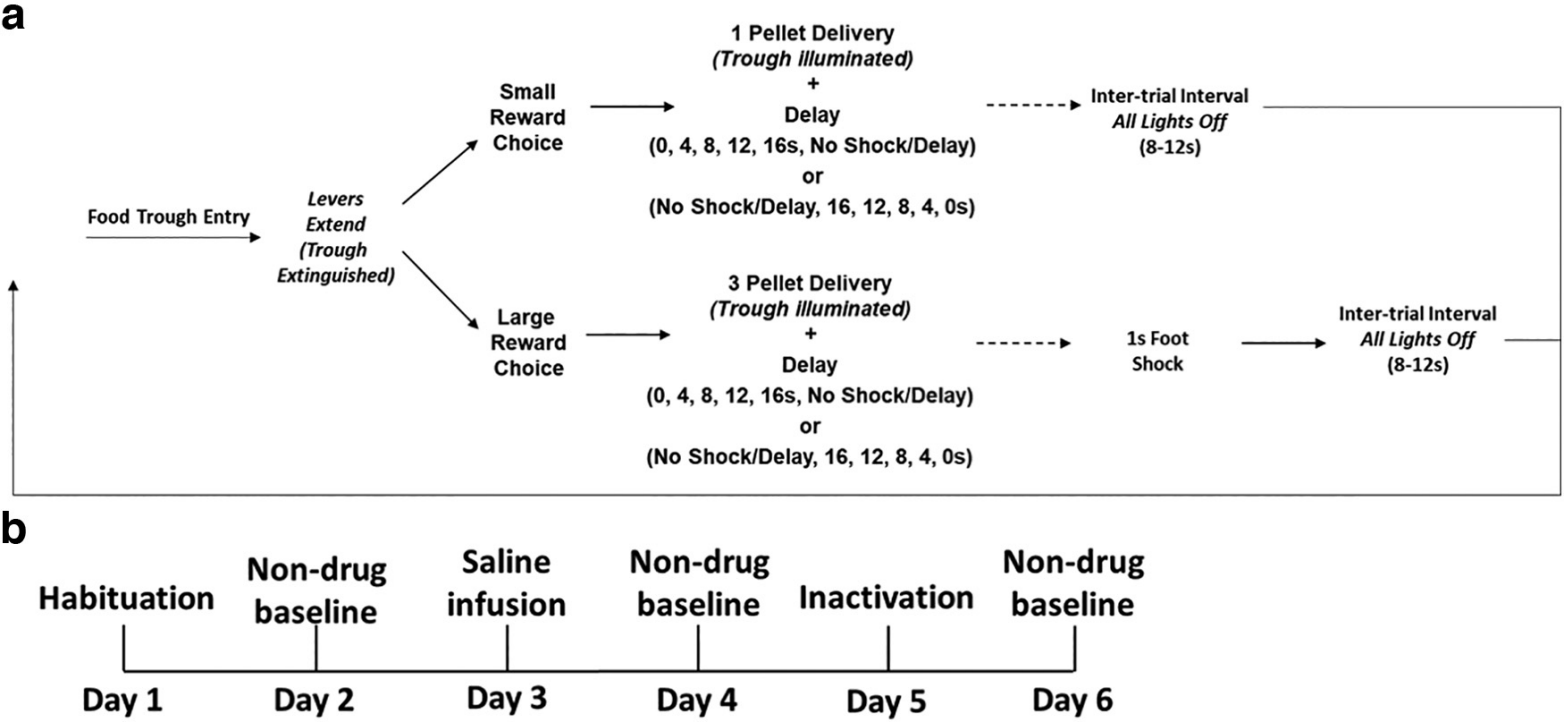
***a***, Delayed punishment decision-making task (DPDT and REVDPDT). Rats chose between two levers, one delivering a one-pellet reward and the other delivering a three-pellet reward accompanied by a delayed foot shock (delay sequence: 0, 4, 8, 12, 16 s, No Shock/Delay for DPDT; No Shock/Delay, 16, 12, 8, 4, 0 s for REVDPDT). ***b***, A six-day microinfusion schedule was used for both brain regions, with inactivation and saline order (days 3 and 5) counterbalanced across subjects.

During DPDT, rats chose between a small reward and larger reward associated with punishment preceded by varying delays. DPDT methodology was comparable to magnitude discrimination described above, with choice between small and large food pellet reinforcers. However, in this task, the large option was associated with a mild, 1-s foot shock. Shock initially occurred immediately after a choice, then systematically took place later in time throughout the task or vice versa (Fig. 1). Lever identity (small or large reward) was fixed within each session and remained consistent across the entirety of training and testing.

Trials began with illumination of the house light and food trough, after which a nose poke into the trough caused one or both levers to extend simultaneously. A press on one lever dispensed a single pellet, while the other dispensed three pellets with a 1-s mild foot shock. After all outcomes were delivered, the house light extinguished, and the next trial proceeded after an ITI of 10 ± 2 s. The sessions were divided into six blocks, with two forced choice and 10 free choice trials in each block for a total of 72 trials. The first two trials of each block were “forced choice” trials in which only a single lever was available to establish the reward/punishment parameters within that block. The following 10 trials were “free-choice” trials in which both levers extended, allowing rats to choose a preferred lever. During the first block, shock occurred immediately after lever press. In each subsequent block, a delay was introduced preceding shock that extended from 0, 4, 8, 12, and 16 s, followed by a block with and No Shock/Delay (Fig. 1). Notably, on trials in which the unpunished lever was chosen, the ITI increased by a period equivalent to the delay preceding shock in that block (4, 8, 12, 16 s) to maintain consistency of trial length regardless of choice.

Shock intensity began at 0.05 mA, then increased by 0.02, 0.03, 0.05, or 0.1 mA (based on sensitivity) in subsequent sessions if rats completed >85% of trials. This incremental increase in shock intensity limited omissions and allowed rats to acquire task parameters. Upon reaching the final shock intensity, subjects trained until they achieved stability, which consisted of no more than a 10% overall shift in daily choice behavior for 2–3 d. To minimize individual differences in performance, shock intensity was titrated for each individual rat until (1) mean choice of punishment across the entire session was between floor (0%) and ceiling (100%), and (2) either a positive slope (DPDT) or negative slope (REVDPDT) was observed for the percent choice of the punished lever. Between these two criteria, all subjects produced a robust discounting curve with sufficient parametric space for treatment to increase or decrease choice of delayed punishment.

Trials were recorded as “omissions” in two possible scenarios: (1) the rat failed to nose poke into the lit port to begin the trial during the allotted 10 s, or (2) the rat nose poked to make the levers extend but did not choose a lever in the allotted 10 s. Omissions could also occur during forced choice trials, but data included in results are restricted to omissions of free choice trials. If a subject omitted a full block of trials, that was tabulated as 10 omissions, and the missing data point was extrapolated using the linear slope of the other data points. We chose a linear slope based on patterns of decision-making observed by Liley et al. (2019). Notably, there was only one session each with missing data for LOFC REVDPDT and BLA DPDT, and no missing points of for LOFC DPDT and BLA REVDPDT, so the amount of extrapolated data were minimal. If two full blocks of trials were omitted in a single session, that session was repeated after an additional baseline session to avoid overuse of extrapolated data. This occurred for one rat in LOFC DPDT, four rats in LOFC REVDPDT, one rat in BLA DPDT, and zero rats in BLA REVDPDT.

### DPDT with descending delays (REVDPDT)

To confirm that effects of inactivation were not driven by task design or aberrant behavioral flexibility, a subset of subjects was trained in a reversed task (REVDPDT). This task was identical to DPDT, except the delays were presented in descending order, beginning with No Shock/Delay, 16, 12, 8, 4, then 0 s preceding foot shock (Fig. 1). Criteria for stability and shaping procedures were comparable to those used for DPDT.

### LOFC and BLA inactivation

After rats reached stable performance in DPDT or REVDPDT, they underwent habituation sessions to acclimate to infusion procedure handling. They then received bilateral drug microinfusions to inactivate either bilateral LOFC or BLA. A drug cocktail of GABA agonists baclofen (Reis and Duarte, 2006) and muscimol (Chandra et al., 2010) dissolved in sterile saline (concentration: 250 ng/μl, 0.5 μl infusion volume over 1 min; Piantadosi et al., 2017; Orsini et al., 2018) was administered into each hemisphere via an automated infusion pump and two 50-μl Hamilton syringes. Behavioral testing commenced after a 15-min absorption period. After a day of baseline testing with no treatment, subjects were given bilateral sterile saline microinfusions (5 μl infused at 0.5 μl/min). Drug/saline order was counterbalanced across subjects.

### Histology

Rats were euthanized with Euthasol, and perfusions were conducted with saline and 10% formalin solution. Brains were extracted, stored in 10% formalin solution, sliced at 60–100 μm using a Cryostat, and mounted onto slides. Cannula placements and infusion localization were confirmed via light microscopy (Paxinos and Watson, 2013).

### Experimental design and statistical analysis

Custom-made MATLAB scripts were used to compile behavioral data, and all statistical analyses were conducted using IBM SPSS Statistics 24. If Mauchly’s test of sphericity was violated, Greenhouse–Geisser values and degrees of freedom were used accordingly. If a rat failed to make any choices during a block of the task, the slope of that subject’s curve was used to extrapolate that missing data point. If two or more blocks of behavioral data were missing, that rat was removed from analysis because of excessive omissions.

Following task acquisition, stable decision-making for DPDT and REVDPDT were measured using a day × block repeated measures ANOVA, quantified as lack of effect of day and a significant effect of block. Effects of microinfusions on behavior were analyzed via sex × infusion (drug vs saline) × block ANOVA. Latency to lever press during testing was evaluated using a mixed sex × safe versus punished lever ANOVA.

## Results

### DPDT/REVDPDT acquisition and stability

The mean number of days to complete shaping procedures before DPDT (magazine training, FR1 for both levers, nose poke, and magnitude discrimination training) was 8.63 for females (*n* = 7) and 7.00 for males, with no significant difference between sexes (*t*_(24)_ = 1.24, *p *=* *0.229). Following shaping, females required significantly more training sessions to reach stability on DPDT (female mean = 42.88 d, male mean = 15.67 d; *t*_(24)_ = 3.63, *p *<* *0.001). After successful training, there were significant effects of block for all subjects in DPDT, such that subjects shifted choice preference from the safe reward to the punished reward as punishment delay increased (LOFC group: *F*_(2.159,21.587)_ = 29.304, *p *<* *0.001; BLA group: *F*_(2.583,28.409)_ = 13.524, *p *<* *0.001). There were no significant differences between sexes in DPDT performance (LOFC: *F*_(5,50)_ = 0.627, *p *=* *0.680; BLA group: *F*_(5,55)_ = 2.204, *p *=* *0.067).

A separate group of rats trained in REVDPDT, in which punishment delays were presented in descending instead of ascending order (Fig. 1). There was no difference in length of shaping for REVDPDT between males and females (female mean = 9.27 d, male mean =7.61 d; *t*_(27)_ = 1.62, *p *=* *0.117). Unlike in DPDT, there were no sex difference in sessions required to achieve stability for REVDPDT (female mean = 30.36 d, male mean = 30.67 d; *t*_(27)_ = 0.03, *p* = 0.976). After task acquisition, there were significant effects of block (LOFC group: *F*_(2.716,27.164)_ = 18.059, *p *<* *0.001; BLA group: *F*_(5,45)_ = 11.075, *p *<* *0.001), such that rats shifted choice away from the punished option as punishment became less delayed/more proximal to the action. There were no sex differences in REVDPDT (LOFC group: *F*_(5,50)_ = 0.657, *p *=* *0.657; BLA group = (*F*_(5,45)_ = 1.191, *p *=* *0.329).

A two-way ANOVA was performed to observe the impact of sex and task (DPDT vs REVDPDT) on differences in titration of foot shock mA (0.05–0.55). There was no significant difference between males and females, although there was a trend toward males having higher terminal shock levels (*F*_(1,53)_ = 2.757, *p *=* *0.10; male DPDT: 0.28 mA; male REVDPDT: 0.30 mA; female DPDT: 0.24 mA; female REVDPDT: 0.27 mA).

### Effects of LOFC inactivation on DPDT

**Figure 2.**
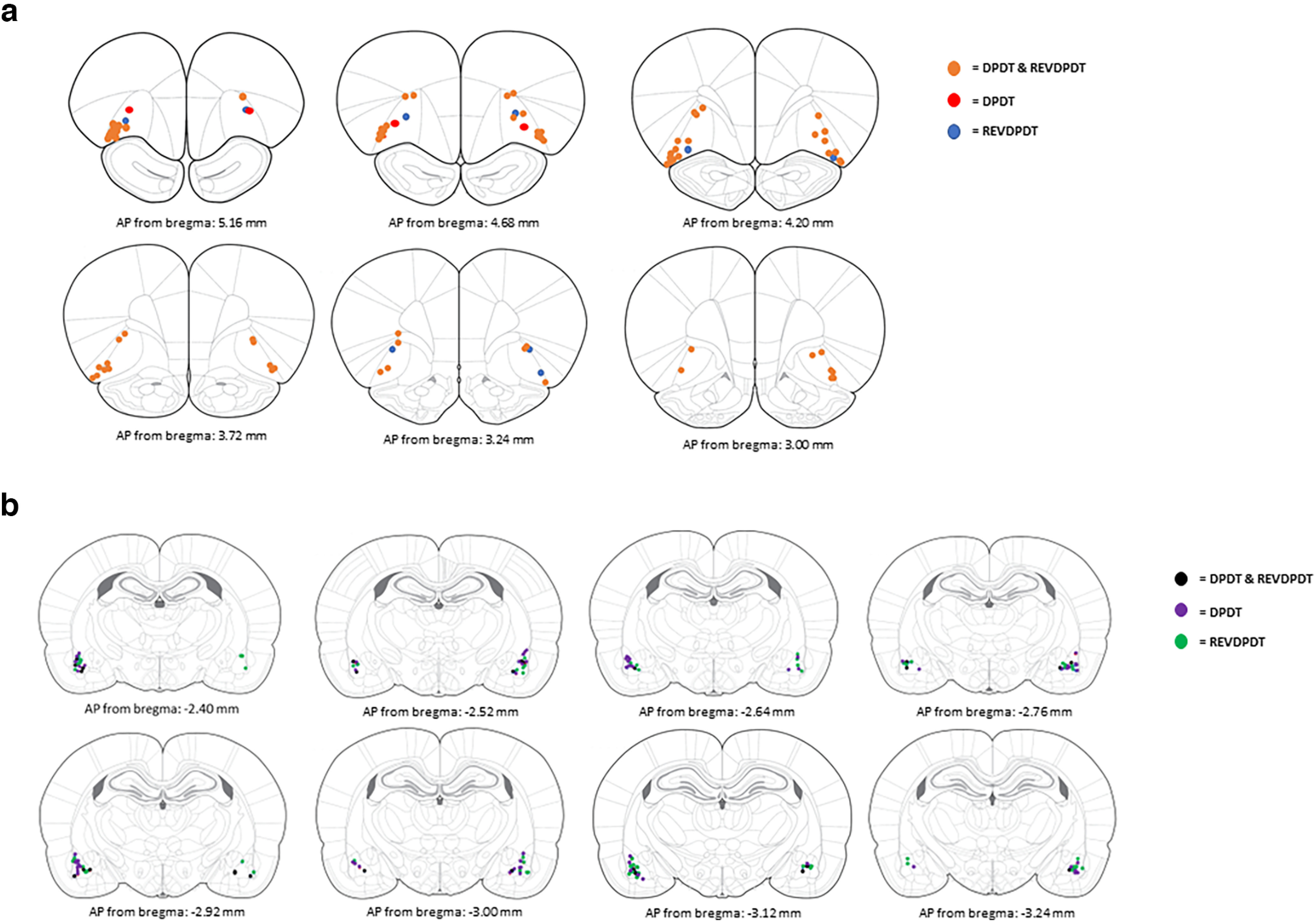
Histologic confirmation of cannulae placements in Lateral orbitofrontal cortex (***a***) and basolateral amygdala (***b***).

**Figure 3.**
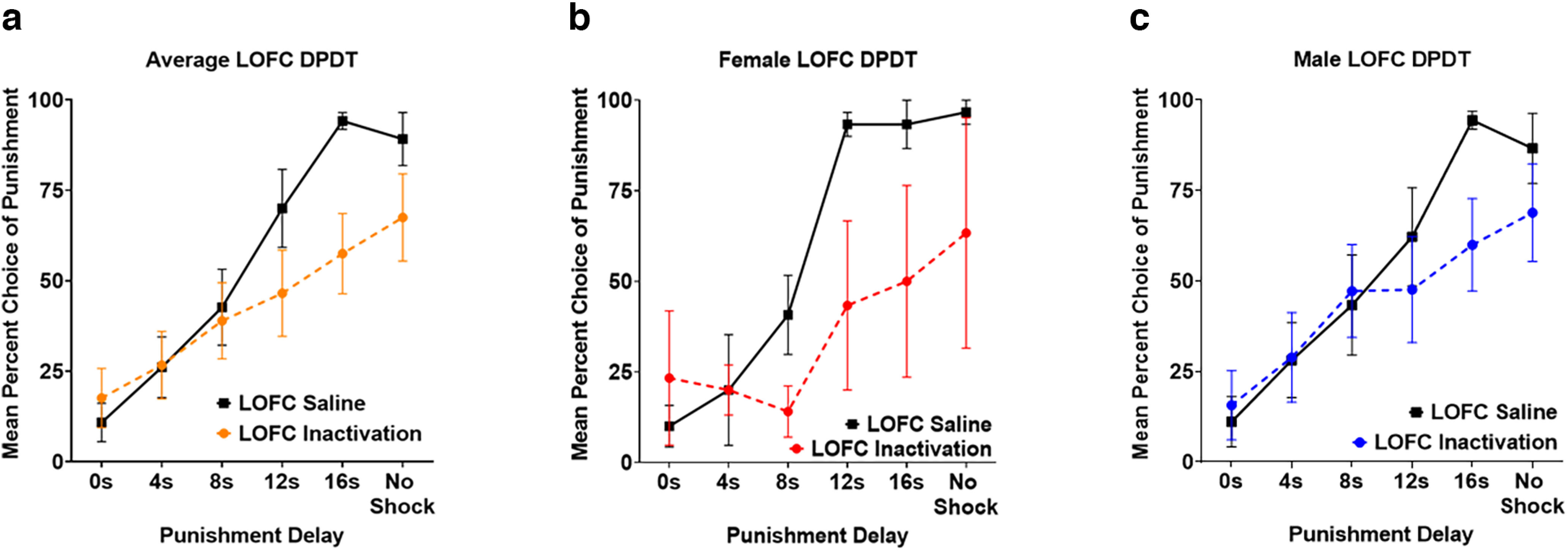
***a***, Lateral orbitofrontal cortex (LOFC) inactivation reduced choice of rewards with delayed punishment without affecting choice of immediate or short-delay punishment. ***b***, ***c***, Females and males showed comparable reduction in choice of delayed but not immediate punishment after LOFC inactivation. All panels display data as mean ± standard error of the mean (SEM). See Extended Data Figure 3-1.

We assessed the effects of acute pharmacological LOFC inactivation on sensitivity to delayed punishment before DPDT using nine male and three female rats, with bilateral cannulae placement in LOFC confirmed before any analyses (Fig. 2*a*). There was a main effect of block (*F*_(2.793,27.931)_ = 26.736, *p *<* *0.001; Fig. 3*a*), such that rats chose the punished reward more frequently when punishment was delayed. There was no effect of sex (*F*_(1,10)_ = 0.018, *p *=* *0.897), inactivation × sex interaction (*F*_(1,10)_ = 1.024, *p *=* *0.335), or inactivation × block × sex interaction (*F*_(5,50)_ = 0.663, *p *=* *0.653), so males and females were merged for all analyses (Fig. 3*b*,*c*).

10.1523/ENEURO.0170-22.2022.f3-1 Extended data Figure 3-1 Statistics summarizing effects of LOFC and BLA inactivation on decision-making with immediate vs. delayed punishment. Data for these analyses are visualized in figures 3,5,7, and 9. Download Figure 3-1, DOC file.

OFC inactivation reduced overall choice of the punished reward (*F*_(1,10)_ = 5.888, *p *=* *0.036). Critically, there was also an inactivation × block interaction (*F*_(5,50)_ = 3.261, *p *=* *0.013; Fig. 3*a*), such that LOFC inactivation only reduced large reward choice when punishment occurred after long delays, but not when punishment occurred immediately or after a short delay. Further investigation using two-tailed paired samples *t* tests (see Table 1) revealed no effects of inactivation in the first three blocks, a near significant difference between drug versus saline for the 12-s delayed shock (*p *=* *0.074), and a significant difference for the 16-s delayed shock (*p *=* *0.005). Finally, there was no effect of LOFC inactivation during the final, unpunished block (*p *=* *0.16), suggesting that LOFC inactivation did not cause gross motivational deficits or inability to discriminate reward magnitude.

**Table 1:** *t* test results comparing average selection of the punished lever following inactivation versus saline during lateral orbitofrontal cortex (LOFC) DPDT (*a*) and *t* test results comparing average selection of the punished lever following inactivation versus saline during LOFC REVDPDT (*b*)

	LOFC				
*a*	Delay	Mean differences	*t*	df	*p*
	0 s	6.76	0.894	11	0.39
				
	4 s	0.56	0.083	11	0.935
				
	8 s	−3.75	−0.516	11	0.616
				
	12 s	−23.43	−1.971	11	0.074
				
	16 s	−36.67	−3.552	11	0.005*
				
	No Shock	−21.67	−1.516	11	0.158

*b*	Delay	Mean differences	*t*	df	*p*
	No Shock	−11.79	−1.863	13	0.085
				
	16 s	−25.56	−2.816	13	0.015*
				
	12 s	−16.39	−1.34	13	0.203
				
	8 s	−9.75	−0.821	13	0.426
				
	4 s	−7.08	−0.582	13	0.571
				
	0 s	−3.36	−0.392	13	0.701
					

Mean difference represents saline mean - inactivation mean.

**Figure 4.**
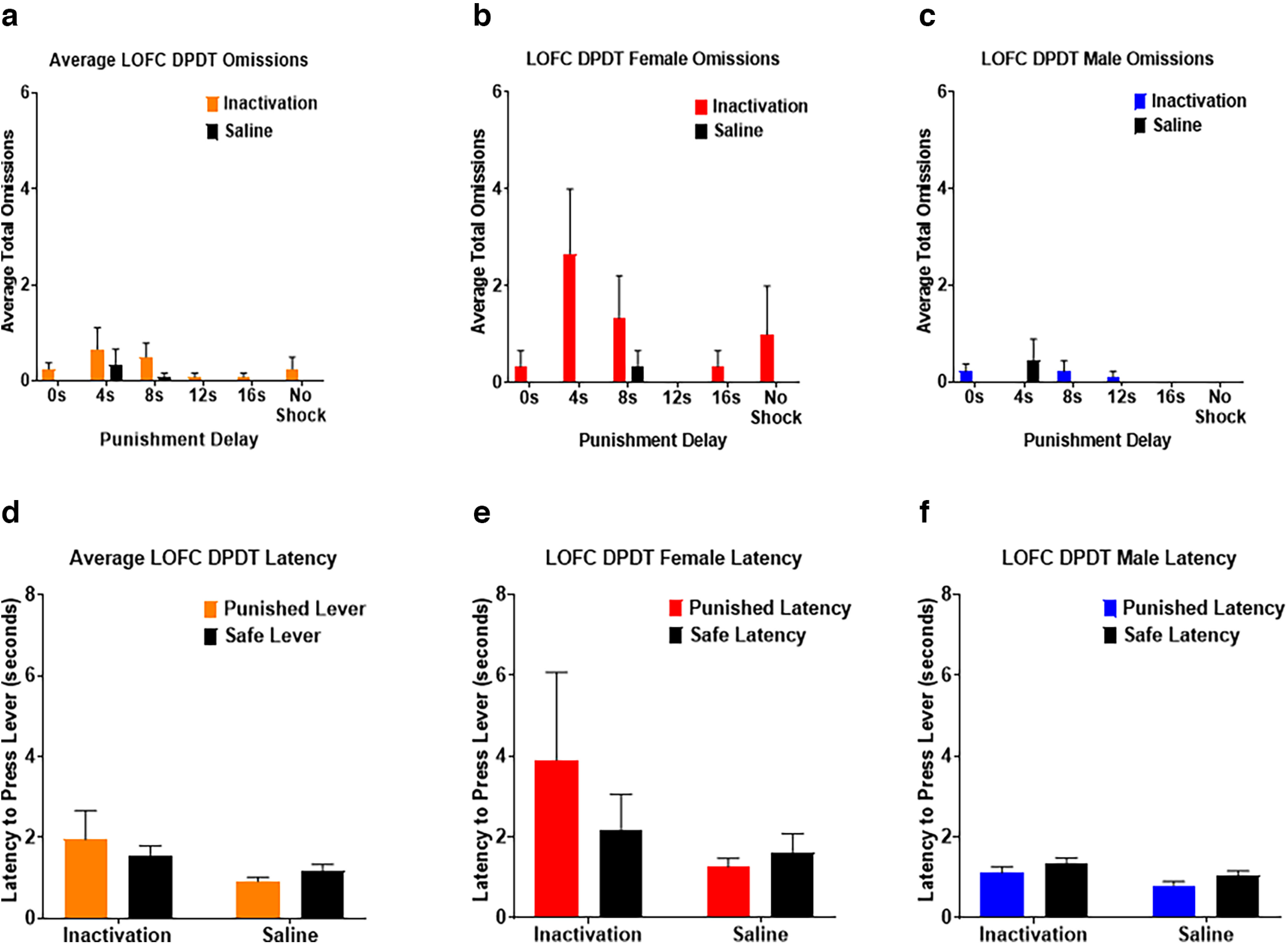
***a***, Lateral orbitofrontal cortex (LOFC) inactivation increased free choice omissions compared with saline, and omissions were most prevalent when punishment occurred after shorter delays. ***b***, ***c***, Females displayed more free choice omissions throughout the task than males. ***d***, LOFC infusions did not affect latency to choose either lever. ***e***, ***f***, Females took longer to choose the punished lever after LOFC inactivation than males. All panels display data as mean ± SEM. See Extended Data Figures 4-1 and 4-2.

Next, we assessed the effects of LOFC inactivation on omitted trials during DPDT. There was a main effect of inactivation (*F*_(1,10)_ = 10.494, *p *=* *0.009; Fig. 4*a*) such that omissions were greater following inactivation compared with saline infusions. There was also an effect of block (*F*_(1.706,17.061)_ = 5.734, *p *=* *0.015), with subjects omitting more trials early in the session wherein punishment had shorter delay times. There was no inactivation × block interaction (*F*_(1.590,15.901)_ = 2.220, *p *=* *0.148). There was a trend toward a main effect of sex in which females displayed more omissions throughout the task than males (*F*_(1,10)_ = 4.395, *p *=* *0.062; Fig. 4*b*,*c*). There was also an inactivation × sex interaction (*F*_(1,10)_ = 9.655, *p *=* *0.011), with females showing increased omitted trials after LOFC inactivation compared with males.

10.1523/ENEURO.0170-22.2022.f4-1 Extended data Figure 4-1 Statistics summarizing effects of LOFC and BLA inactivation on trial omissions. Data for these analyses are visualized in figures 4,6,8, and 10. Download Figure 4-1, DOC file.

10.1523/ENEURO.0170-22.2022.f4-2 Extended data Figure 4-2 Statistics summarizing effects of LOFC and BLA inactivation on choice latency. Data for these analyses are visualized in figures 4,6,8, and 10. Download Figure 4-2, DOC file.

We next investigated the effects of LOFC inactivation on latency to choose a lever. There was no significant difference in latency to choose safe versus punished levers (*F*_(1,11)_ = 0.073, *p *=* *0.792; Fig. 4*d*), nor was there an effect of LOFC inactivation *(F*_(1,11)_ = 0.003, *p *=* *0.960). However, there was a trend toward an inactivation × lever type interaction (*F*_(1,11)_ = 4.435, *p *=* *0.059), such that inactivation lengthened the time required for subjects to choose the punished but not safe lever. There was also an effect of sex (*F*_(1,11)_ = 8.871, *p *=* *0.013; Fig. 4*e*,*f*), with females taking longer than males to make a choice, but no sex × lever type interaction (*F*_(1,11)_ = 2.319, *p *=* *0.156).

### Effects of LOFC inactivation on REVDPDT

**Figure 5.**
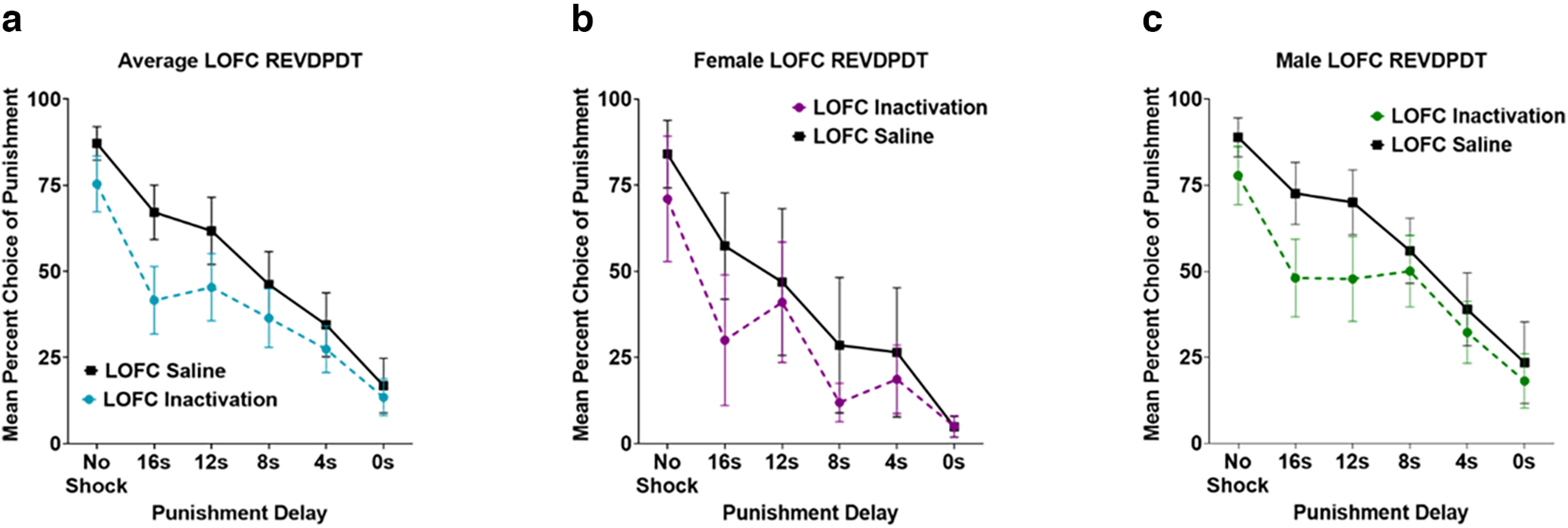
***a***, Inactivation of Lateral orbitofrontal cortex (LOFC) during REVDPDT shifted choice away from the punished reward as delays decreased. ***b***, ***c***, Females and males displayed similar reduction in choice of the punished lever compared with safe when delays decreased during LOFC inactivation. All panels display data as mean ± SEM. See Extended Data Figure 3-1.

To test that the effects observed with DPDT were not solely because of inflexible behavior (leading to a “flattened” discounting curve), we trained rats in a reversed version of DPDT (REVDPDT) with descending delays preceding punishment (blocks: No Shock, 16, 12, 8, 4, 0 s). Analysis of brain sections determined that five female and nine male rats (*n* = 14) had accurate cannula placement in LOFC (Fig. 2*a*). Importantly, as with DPDT, there was a significant main effect of block (*F*_(5,60)_ = 21.468, *p *<* *0.001; Fig. 5*a*), such that rats shifted choice away from the punished reward as delay decreased. There was no effect of sex (*F*_(1,12)_ = 3.119, *p *=* *0.103), inactivation × sex interaction (*F*_(1,12)_ = 0.002, *p *=* *0.961), or inactivation × block × sex interaction (*F*_(5, 60)_ = 0.381, *p *=* *0.860), so males and females were again merged for subsequent analyses (Fig. 5*b*,*c*).

There were no effects of inactivation on choice of the punished option (*F*_(1,12)_ = 1.920, *p *=* *0.191), nor an inactivation × block interaction (*F*_(5,60)_ = 1.128, *p *=* *0.355). However, based on the LOFC inactivation exerting the most substantial effects during the 16-s delayed punishment in the standard DPDT (Fig. 5*a*), we probed for an effect of inactivation on this block during REVDPDT. We observed that LOFC inactivation did indeed reduce choice of the punished reward in this block (*t*_(13)_ = −2.816, *p *=* *0.015), with no differences observed in other blocks (Table 1). This revealed that, as in the original task, LOFC inactivation reduced choice of the punished reward when punishment occurred after a long (16-s) delay, but not when punishment coincided with choice after shorter (0- to 12-s) delays.

**Figure 6.**
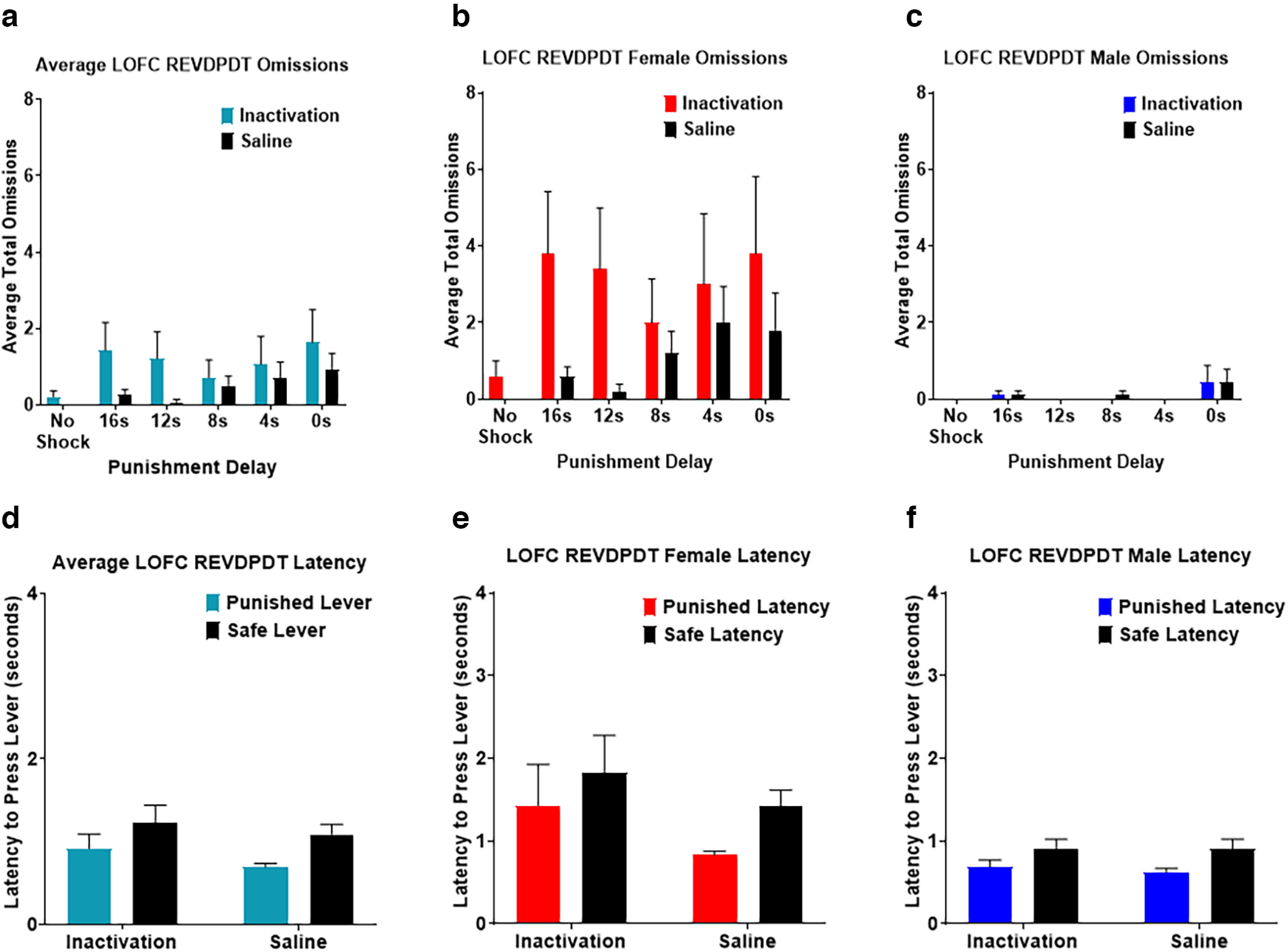
***a***, Inactivation of Lateral orbitofrontal cortex (LOFC) during REVDPDT increased free choice omissions during inactivation compared with saline. ***b***, ***c***, Females omitted more free choice trials than males throughout the task. ***d***, There were no differences in latency to choose either the punished or safe levers for both inactivation and saline infusions during REVDPDT. ***e***, ***f***, Females required more time to select the punished lever than males. All panels display data as mean ± SEM. See Extended Data Figures 4-1 and 4-2.

We next investigated the effects of LOFC inactivation on omissions during REVDPDT. This revealed a near significant effect of inactivation (*F*_(1,12)_ = 4.690, *p *=* *0.051), such that LOFC inactivation increased omissions compared with saline. There was also a significant effect of block (*F*_(2.363,28.355)_ = 4.277, *p *=* *0.019) with omissions increasing after the first, unpunished block (Fig. 6*a*). There was no inactivation × block interaction (*F*_(2.760,33.122)_ = 2.160, *p *=* *0.116). There was an effect of sex (*F*_(1,12)_ = 11.974, *p *=* *0.005), with females omitting more trials than males. There was also an inactivation × sex interaction (*F*_(1,12)_ = 4.887, *p *=* *0.047), with females, but not males, demonstrating an increase in omissions after LOFC inactivation (Fig. 6*b*,*c*).

Finally, we examined the effects of LOFC inactivation on response latency. There was no effect of inactivation (*F*_(1,11)_ = 3.403, *p *=* *0.092) or inactivation × lever type interaction (*F*_(1,11)_ = 0.300, *p *=* *0.595; Fig. 6*d*). There was a difference in time taken to choose a lever (*F*_(1,11)_ = 11.619, *p *=* *0.006). There was also a main effect of sex (*F*_(1,11)_ = 11.420, *p *=* *0.006; Fig. 6*e*,*f*) such that females were slower to respond than males. However, there were no sex × lever type (*F*_(1,11)_ = 1.911, *p *=* *0.194) or sex × drug interactions (*F*_(1,11)_ = 2.745, *p *=* *0.126).

### Effects of BLA inactivation on DPDT

**Figure 7.**
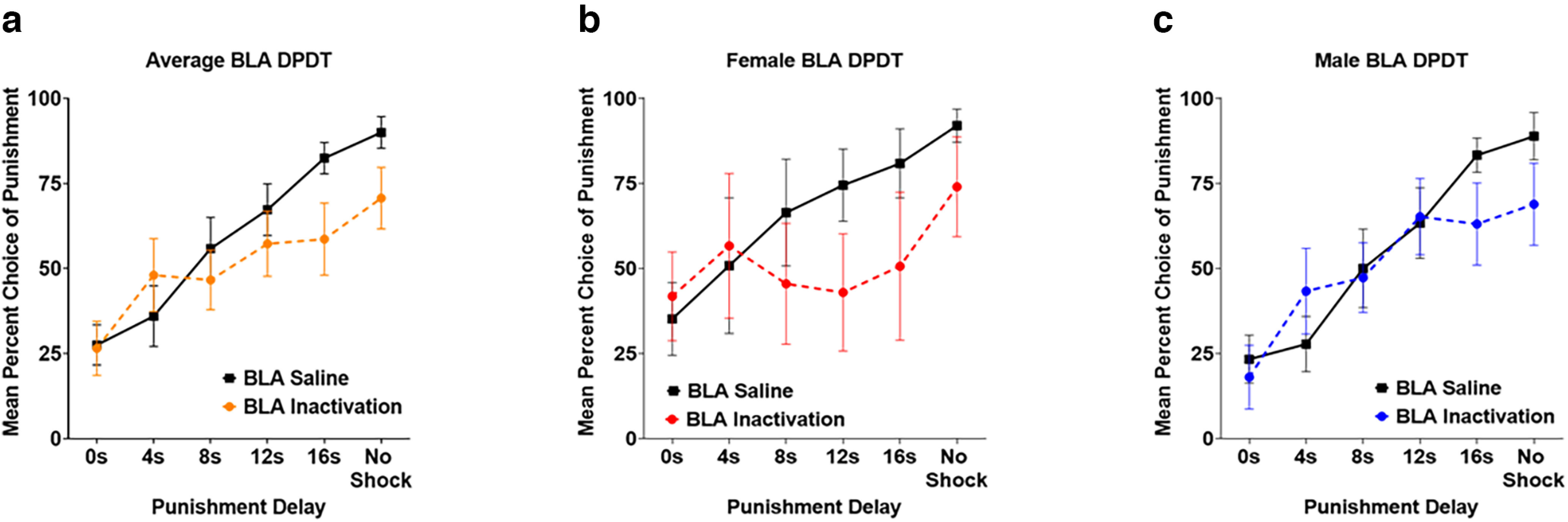
***a***, Basolateral amygdala (BLA) inactivation did not affect choice when punishment was immediate, but reduced choice of the punished reward when delays were longer. ***b***, ***c***, Females and males both displayed reduction in choice of delayed punishment but not immediate punishment following BLA inactivation. All panels display data as mean ± SEM. See Extended Data Figure 3-1.

Analysis of brain sections determined that five female and nine male rats (*n* = 14) had accurate cannula placement (Fig. 2*b*). As previously, there was a significant effect of block (*F*_(5,60)_ = 16.312, *p *<* *0.001; Fig. 7*a*) indicating that rats selected the punished reward more frequently when punishment was delayed. There was no main effect of sex (*F*_(1,12)_ = 0.231, *p *=* *0.639), inactivation × sex interaction (*F*_(1,12)_ = 0.420, *p *=* *0.529), block × sex (*F*_(5,60)_ = 1.479, *p *=* *0.210), or inactivation × block × sex interaction (*F*_(5,60)_ = 1.093, *p *=* *0.374), so sexes were combined for all analyses (Fig. 7*b*,*c*).

There was no overall main effect of inactivation on choice (*F*_(1,12)_ = 1.800, *p *=* *0.205). However, there was an inactivation × block interaction (*F*_(5,60)_ = 3.102, *p *=* *0.015) such that BLA inactivation reduced choice of the punished reward when punishment was delayed (Fig. 7*a*) but did not affect choice when punishment was immediate. Paired samples *t* tests revealed this significant reduction in choice of the punished reward only occurred in the 16-s delay condition (*t*_(13)_ = −2.787, *p *=* *0.015; Table 2). Interestingly, BLA inactivation also reduced choice of the large reward in the final, unpunished block (*t*_(13)_ = −3.006, *p *=* *0.010).

**Table 2:** *t* test results comparing average selection of the punished lever following inactivation versus saline during Basolateral amygdala (BLA) DPDT (*a*) and *t* test results comparing average selection of the punished lever following inactivation versus saline during BLA REVDPDT (*b*)

	BLA				
*a*	Delay	Mean difference	*t*	df	*p*
	0 s	−1.01	−0.125	13	0.902
				
	4 s	12.07	1.247	13	0.234
				
	8 s	−9.23	−0.775	13	0.452
				
	12 s	−10.05	−0.863	13	0.404
				
	16 s	−23.81	−2.787	13	0.015*
				
	No Shock	−19.29	−3.006	13	0.010*

*b*	Delay	Mean difference	*t*	df	*p*
	No Shock	−7.33	−0.929	14	0.369
				
	16 s	−7.09	−1.245	14	0.234
				
	12 s	−9.46	−1.049	14	0.312
				
	8 s	−1.09	−0.113	14	0.912
				
	4 s	−3.23	−0.285	14	0.78
				
	0 s	−3.28	−0.382	14	0.708

Both include mean differences between BLA inactivation and saline. Mean difference represents saline mean - inactivation mean.

**Figure 8.**
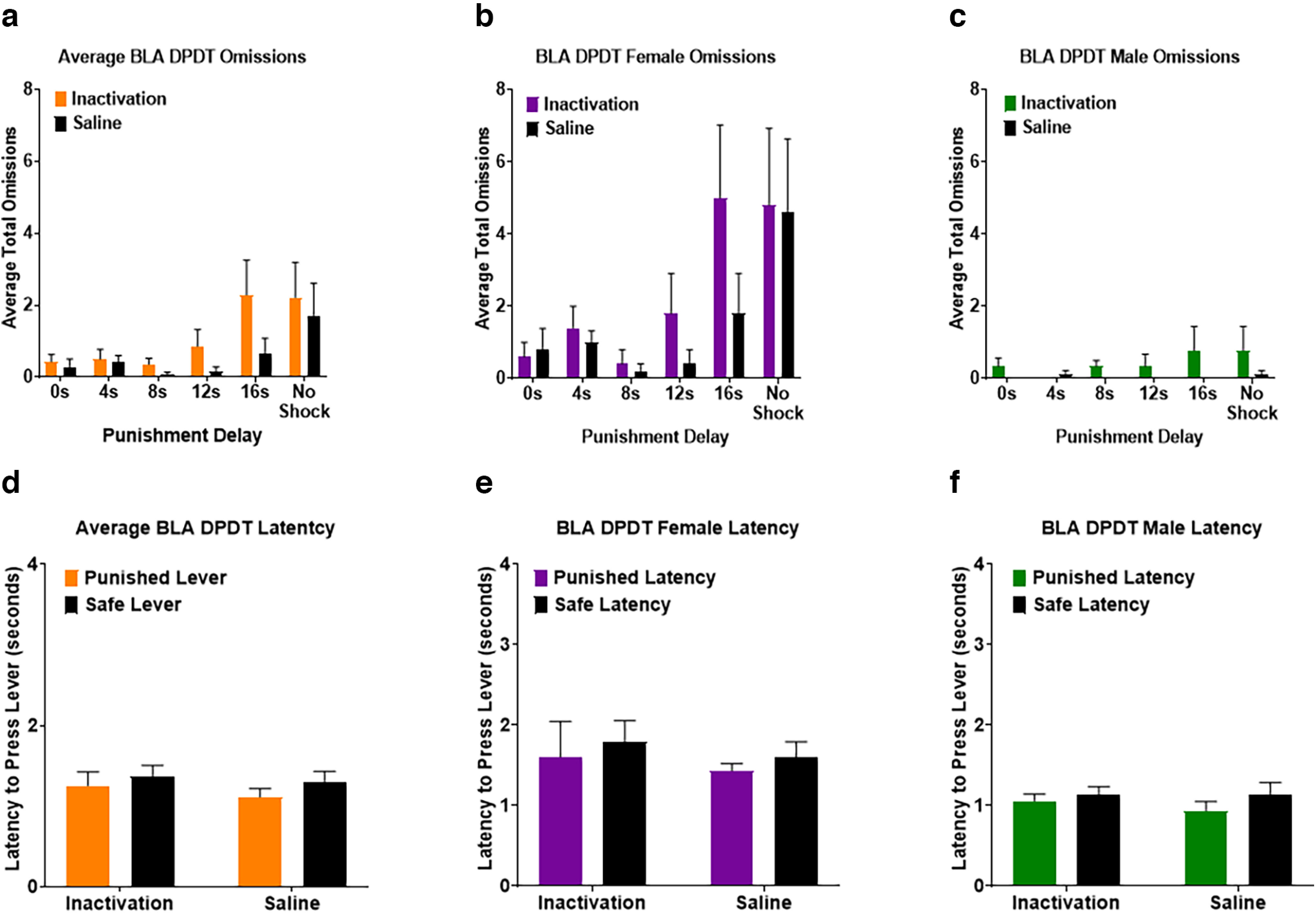
***a***, Subjects increased free choice omissions during longer delay times of DPDT following Basolateral amygdala (BLA) inactivation compared with saline. ***b***, ***c***, Females had more free choice omissions than males throughout the task. ***d***, There were no effects of inactivation or saline on latency to choose between the punished and safe levers. ***e***, ***f***, Females required more time to choose a lever than males. All panels display data as mean ± SEM. See Extended Data Figures 4-1 and 4-2.

There was an effect of block on omissions, such that subjects increased omissions as the session progressed (*F*_(1.260,15.118)_ = 8.647, *p *=* *0.007; Fig. 8*a*). While there was no overall effect of BLA inactivation on omissions (*F*_(1,12)_ = 3.470, *p *=* *0.087), there was a near-significant inactivation × block interaction (*F*_(2.356,28.276)_ = 3.095, *p *=* *0.053), with BLA inactivation causing a greater increase in omissions as the task continued (Fig. 8*a*). Additionally, there was an effect of sex in that females had more omissions than males (*F*_(1,12)_ = 9.555, *p *= 0.009), but no inactivation × sex interaction (*F*_(1,12)_ = 0.502, *p *=* *0.492) or inactivation × block × sex interaction (*F*_(5,60)_ = 1.891, *p *= 0.109; Fig. 8*b*,*c*).

We next investigated the effects of BLA inactivation on decision latency. There was no main effect of inactivation (*F*_(1,12)_ = 1.339, *p *=* *0.270), latency between punished versus safe levers (*F*_(1,12)_ = 0.770, *p *=* *0.397), or inactivation × lever type interaction (*F*_(1,12)_ = 0.178, *p *=* *0.680; Fig. 8*d*). There was a main effect of sex (*F*_(1,12)_ = 12.551, *p *=* *0.004), with females taking longer to make a choice than males; but no sex × lever type interaction (*F*_(1,12)_ = 0.223, *p *=* *0.645) or sex × inactivation interaction (*F*_(1,12)_ = 0.016, *p *=* *0.903; Fig. 8*e*,*f*).

### Effects of BLA inactivation on REVDPDT

**Figure 9.**
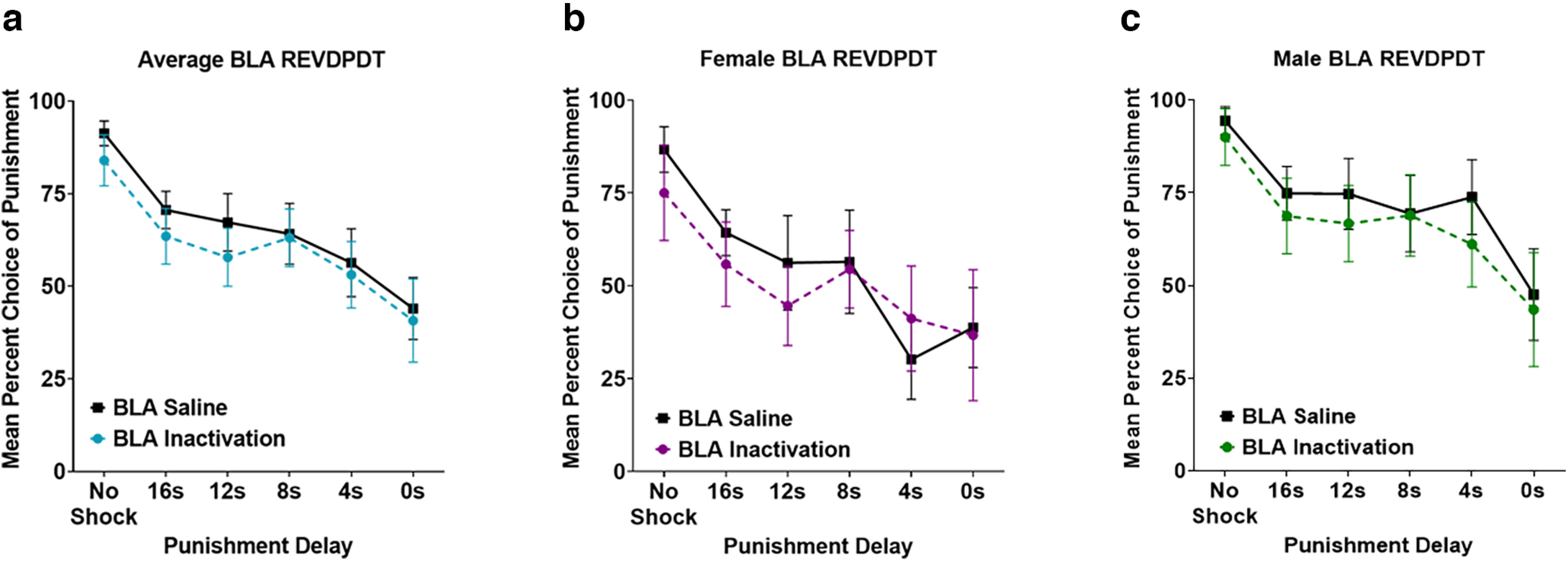
***a***, Basolateral amygdala (BLA) inactivation did not affect decision-making during REVDPDT. ***b***, ***c***, Neither females nor males were affected by BLA inactivation. All panels display data as mean ± SEM. See Extended Data Figure 3-1.

BLA was inactivated before REVDPDT in 6 female and 9 male rats (Fig. 2*b*). As expected, there was a main effect of block (*F*_(5,65)_ = 12.065, *p *<* *0.001; Fig. 9*a*) such that the initial preference for the large reward shifted toward the safe reward as punishment delay decreased. Similar to DPDT, there was no main effect of sex (*F*_(1,13)_ = 2.489, *p *=* *0.139), sex × inactivation interaction (*F*_(1,13)_ = 0.015, *p *=* *0.905), block × sex interaction (*F*_(5,65)_ = 1.005, *p *= 0.422), or inactivation × block × sex interaction (*F*_(5,65)_ = 0.905, *p *=* *0.483; Fig. 9*b*,*c*), so sexes were again combined for analyses.

There was no effect of inactivation (*F*_(1,13)_ = 0.444, *p *= 0.517) or inactivation × block interaction (*F*_(5,65)_ = 0.427, *p *=* *0.828; Fig. 9*a*) on choice of the punished reward. There were also no effects of inactivation for any block (*p *>* *0.05; Table 2), suggesting that unlike standard DPDT, BLA inactivation had no effects on REVDPDT with descending punishment delays.

**Figure 10.**
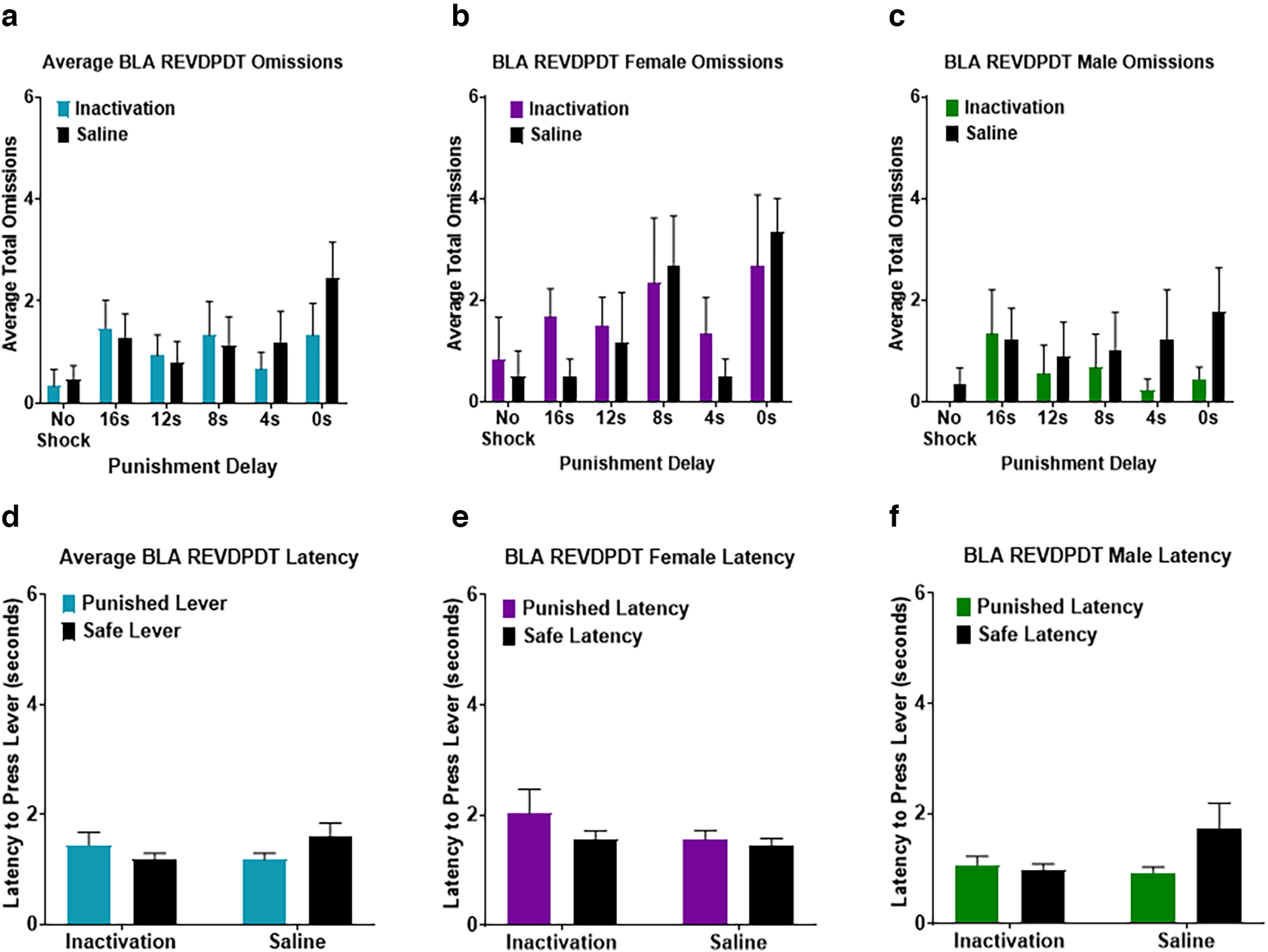
***a***, Basolateral amygdala (BLA) inactivation did not affect free choice omissions during REVDPDT. ***b***, ***c***, Females and males displayed a comparable number of free choice omissions. ***d***, Latency to choose between the punished and safe levers was not affected by inactivation. ***e***, ***f***, Females had greater latency to select a lever than males. All panels display data as mean ± SEM. See Extended Data Figures 4-1 and 4-2.

As with LOFC DPDT and REVDPDT previously, BLA REVDPDT omissions increased throughout the session (*F*_(2.405,31.263)_ = 3.943, *p *=* *0.023; Fig. 10*a*). However, BLA inactivation had no effect on omissions (*F*_(1.000,13.000)_ = 0.070, *p *=* *0.795). There was also no effect of sex (*F*_(1,13)_ = 1.660, *p *=* *0.220), inactivation × block interaction (*F*_(2.199,28.589)_ = 1.082, *p *=* *0.357), inactivation × sex interaction (*F*_(1,13)_ = 0.695, *p *=* *0.419), or inactivation × block × sex interaction (*F*_(5,65)_ = 0.195, *p *=* *0.963; Fig. 10*a–c*).

Effects of BLA inactivation on decision-making latency revealed no main effect of inactivation (*F*_(1,11)_ = 0.003, *p *=* *0.960), difference in choice latency between punished versus safe rewards (*F*_(1,11)_ = 0.073, *p *=* *0.792), or inactivation × lever type interaction (*F*_(1,11)_ = 4.435, *p *=* *0.059; Fig. 10*d*). There was a main effect of sex (*F*_(1,11)_ = 8.871, *p *=* *0.013), such that females took longer to respond than males. There was no sex × lever type interaction (*F*_(1,11)_ = 2.319, *p *=* *0.156) or sex × inactivation interaction (*F*_(1,11)_ = 2.368, *p *=* *0.152; Fig. 10*e*,*f*).

## Discussion

While discounting of delayed rewards has been well-studied, little is known about the neural substates underlying delayed punishment discounting. Here we replicated previous findings that rats undervalue punishment preceded by a delay, reflected as increased choice of rewards with delayed compared with immediate punishment. This increased choice of delayed punishment was comparable between ascending and descending punishment delay schedules. LOFC inactivation reduced choice of delayed rewards with both ascending and descending delays, although the effect was confined to the longest (16-s) punishment delay in the descending condition. BLA inactivation also reduced choice of ascending delayed punishments, but not punishments with descending delays.

### LOFC regulates sensitivity to delayed punishment

LOFC inactivation reduced choice of rewards with longer delayed (but not immediate) punishments, suggesting that LOFC contributes to underestimation of delayed punishment during reward seeking. This is comparable to OFC driving discounting of delayed rewards (Mobini et al., 2002; Rudebeck et al., 2006), although effects of OFC manipulation vary based on task design and individual differences in impulsivity (Winstanley, 2004; Zeeb et al., 2010). Notably, a population of neurons in OFC signals reduction in value of delayed rewards (Roesch et al., 2006); it is possible that OFC activity signals discounting of impending punishment in similar fashion. However, based on the lack of correlation between delay discounting of reward and punishment (Liley et al., 2019), it is feasible that OFC encodes delayed outcomes differently based on motivational valence.

One explanation for reduced choice of delayed punishment after LOFC inactivation is impaired ability to adapt to changes in delay. This inability to update task contingencies would likely manifest as a “flattened” discounting curve. However, this is unlikely based on effects of LOFC inactivation during REVDPDT, in which punishment delays decreased throughout the session. As with standard DPDT, LOFC inactivation reduced choice of delayed punishment but not immediate or briefly delayed punishment, resulting in a “steeper” curve. This verifies that LOFC inactivation does not impair behavioral flexibility in this context. Notably, LOFC inactivation in REVDPDT evoked more selective effects than during the standard task, only reducing choice of punishment during the longest (16-s) delay. Performing individual comparisons typically requires the presence of an interaction; however, because the 16-s delay produced the greatest effect in standard DPDT, there was strong rationale to selectively probe this data point in REVDPDT. Nonetheless, the effects of LOFC inactivation on REVDPDT are not as substantial as on DPDT. Future replications using longer delays may increase the sensitivity of this task to LOFC inactivation and other experimental manipulations.

It is also possible that reduced choice of delayed punishment following LOFC inactivation was not caused by reduced delayed punishment discounting, but by increased overall sensitivity to punishment. However, this is unlikely because LOFC inactivation did not influence choice when punishment was immediate or after a short delay (0–8 s). Another possible explanation for reduced large reward choice is that LOFC inactivation impaired magnitude discrimination, as LOFC has been shown to signal reward value (Van Duuren et al., 2008; Simon et al., 2015; Ballesta and Padoa-Schioppa, 2019). This is unlikely because both DPDT and REVDPDT include a punishment-free 1 versus three pellet block, during which LOFC inactivation did not influence reward choice.

It is possible that rats performing DPDT are not discounting delayed punishment but are instead unaware of impending punishment because of reduced temporal contiguity between action and outcome, leading to increased choice of options with delayed punishment. OFC has a well-established role in outcome representation (Ursu and Carter, 2005; Mainen and Kepecs, 2009; Panayi et al., 2021); therefore, if choice of delayed punishment was driven by reduced punishment expectancy, inactivation of LOFC would further disrupt expectancy and increase choice of delayed punishment. However, LOFC inactivation here had the opposite effect, reducing choice of rewards with delayed punishment. Therefore, the most plausible explanation for the reduced choice of delayed punishment is reduced punishment delay discounting.

While decision-making in DPDT involves delay discounting of punishment, it is important to consider that this is superimposed over reward-based decision-making. Optimal choice in DPDT requires two cognitive processes: merging punishment with expected delay to produce a negative motivational value, and integration of this aversive information with the value of appetitive outcomes (one vs three pellets). Thus, it is difficult to disentangle which of these factors is affected by LOFC inactivation. OFC is theorized to encode a dynamic cognitive map of task space that integrates all available action-outcome contingencies to guide decision-making (Wilson et al., 2014; Schuck et al., 2016; Cai, 2021). It is possible that the ability to incorporate delays preceding punishment into this “map” is dependent on LOFC. More granular research of the behavioral components of this task along with assessment of functional neuronal activity is necessary to fully delineate how LOFC drives decision-making in this context.

A previous study determined that males select rewards accompanied by delayed punishment more than females when shock intensity was comparable for all subjects (Liley et al., 2019). Baseline sex differences in decision-making were not observed here, as shock levels were titrated to avoid ceiling or floor effects for each subject (Orsini and Simon, 2020). Surprisingly, evaluation of final shock intensities per group for LOFC and BLA did not reveal a sex difference, although there was a trend toward males requiring a higher intensity shock than females. It is important to note that there were 17 more males than females overall, which likely contributed to this lack of main effect of sex. This imbalance was caused by several females failing to complete acquisition of the task and proceed to the testing phase, remaining at 0% choice of the large reward even at extremely low shock intensity. Had these subjects been included, it is likely that we would have replicated the sex difference observed by Liley et al. (2019). Regardless, the results of the inactivation experiments suggest that both LOFC and BLA regulate choice in DPDT similarly in both males and females. Because of difficulty with task acquisition, the sample size of females was smaller than males; however, the lack of sex differences in sensitivity to inactivation permitted merging sexes for each experiment.

Female rats omitted more trials than males, consistent with previous data showing that estradiol drives avoidance during punishment-based decision-making (Orsini et al., 2021). Inactivation also increased latency for females to make a choice compared with males. Finally, females required almost three times as many sessions as males to acquire DPDT. This is likely attributable to the first exposure to immediate shock driving avoidance of all options (including safe choice) in females. This subsequently increased the time required for females to be exposed to all task parameters, attenuating the overall rate of task acquisition. Alternatively, when the task began with delayed punishment (REVDPDT), females acquired the task as quickly as males. Therefore, beginning training with the option of immediate punishment may cause females to generalize punishment to both levers and completely disengage from the task early in training.

### BLA inactivation selectively reduces choice of delayed punishment

Like LOFC, BLA inactivation did not reduce choice of rewards with immediate punishment, but decreased selection of longer delayed punishments (16 s). This is somewhat surprising, as BLA has been shown to regulate choice of options associated with immediate punishment. Lesioning BLA caused increased choice of punished rewards during the risky decision-making task (Orsini et al., 2015b) and BLA inactivation reduced the suppression of punished reward seeking responses to a foot shock associated lever (Jean-Richard-dit-Bressel and McNally, 2015). This finding also indicates that BLA inactivation only reduces punishment choice in situations with dynamic punishment delays. The reduced delay discounting of punishment after BLA inactivation is consistent with optical BLA inactivation reducing delay discounting of reward (Hernandez et al., 2019), although BLA lesions increase reward discounting (Winstanley, 2004).

One possible explanation for the lack of effects of BLA inactivation on sensitivity to punishment is the location of infusions within BLA. Jean-Richard-dit-Bressel and McNally (2015) found a functional distinction between anterior and posterior BLA, with only the posterior/caudal subregion being involved with detecting the aversive value of punishment. Our placements were mostly centralized in BLA; nonetheless, it is possible that these were insufficient to fully suppress the function of posterior BLA function. When we separated subjects based on cannula location, we found no statistical difference between subjects with anterior versus posterior placement (data not shown). However, more than half of the subjects were removed from this analysis because of centralized placement; thus, a more directed experiment is required to compare anterior versus posterior BLA function in this context. Regardless, the stereotaxic coordinates used here were sufficient to alter choice of delayed punishment in DPDT, suggesting that sensitivity to delayed punishment BLA may be independent of the anterior/posterior axis.

Interestingly, BLA inactivation reduced choice of delayed punishment during ascending punishment delays (DPDT), but not descending delays (REVDPDT). It is possible that decision-making beginning with the option of immediate punishment creates a “high stress” scenario wherein BLA is recruited to influence choice. Conversely, REVDPDT begins with no possibility of punishment, which may reduce BLA involvement in decision-making. LOFC inactivation also exerted more influence on choice during DPDT than REVDPDT, suggesting that transitioning from delayed punishment to immediate punishment may be less sensitive to neural manipulations than ascending punishment delays. Notably, there was a qualitative difference in saline responding between LOFC and BLA groups in REVDPDT (Figs. 5, 9), suggesting that treatment in the latter experiment may have shifted baseline choice toward the punished reinforcer. However, decision-making after saline was highly comparable to baseline decision-making before any infusions (*p *=* *0.781; data not shown), suggesting that this difference was independent of BLA manipulation.

While BLA inactivation reduced choice of the punished reward at the longest delay length, we also observed that this avoidance persisted after punishment was removed in the final block of DPDT. It is possible that BLA provides information about reward safety that is no longer available after inactivation, leading to avoidance of the large reward regardless of shock presence. This is supported by evidence that BLA contributes to the processing of safety signals during previously punished scenarios (Ng et al., 2018). The perseverative large reward avoidance could also be explained by BLA inactivation impairing ability to adapt to task changes. However, BLA inactivation during REVDPDT had no impact on choice, suggesting that BLA is not critical for flexibility within a session.

There were no sex differences in decision-making following BLA inactivation, but there were sex differences in other behavioral measures. Omissions were greater in females only during DPDT, suggesting that females are prone to omitting trials when sessions begin with immediate shock. Following BLA inactivation, there was a trend toward increased omissions in later trials as the session progressed, which diverged from increased omissions with immediate shock after LOFC inactivation. It is possible that LOFC but not BLA inactivation increased the salience of immediate punishment (as shown by Orsini et al., 2015b), reflected as task disengagement rather than changes in % choice. Notably, these effects were primarily in females, and the BLA group had more females than LOFC (5 vs 3). Additional experiments focused on female subjects will enable deeper investigation of this phenomenon. Finally, as seen with the LOFC experiments, BLA females had longer response latencies than males for both tasks.

The OFC and BLA are densely interconnected (Price, 2007), and both contribute to decision-making informed by reward and punishment (Jean-Richard-Dit-Bressel and McNally, 2016; Orsini et al., 2017). Furthermore, connections between LOFC and BLA are critically involved with encoding/retrieving the incentive value of cues and actions to help guide future decision-making (Groman et al., 2019; Malvaez et al., 2019; Sias et al., 2021). We observed that both LOFC and BLA appear to subserve similar roles in delay discounting of punishment, as inactivation of either region reduced choice of delayed punishment. It is possible that BLA-LOFC work in concert to regulate delayed punishment discounting. However, based on the selective effects of BLA inactivation on DPDT but not REVDPDT, BLA may only be engaged in situations beginning with the threat of immediate punishment. Future experiments will investigate the specific role of this circuit in sensitivity to delayed versus immediate punishment.

In conclusion, insensitivity to delayed punishment is a critical aspect of psychiatric illnesses, during which future consequences are often undervalued in favor of immediate rewards. To our knowledge, this is the first assessment of the neurobiological mechanisms underlying this critical phenotype. These data indicate that LOFC and BLA circuitry may serve as a promising therapeutic target to improve sensitivity to delayed punishment during decision-making.

## Synthesis

Reviewing Editor: Laura Bradfield, University of Technology Sydney Decisions are customarily a result of the Reviewing Editor and the peer reviewers coming together and discussing their recommendations until a consensus is reached. When revisions are invited, a fact-based synthesis statement explaining their decision and outlining what is needed to prepare a revision will be listed below. The following reviewer(s) agreed to reveal their identity: Matthew Gardner. Note: If this manuscript was transferred from JNeurosci and a decision was made to accept the manuscript without peer review, a brief statement to this effect will instead be what is listed below.

Overall the reviewers found the manuscript to contain important novel findings regarding the role of the lateral orbitofrontal cortex (lOFC) in delay discounting punishments. However, there are some issues with the methodology and statistical analyses that need to be addressed. Please see address the specific points in the reviews that follow:

Reviewer #1

Regarding statistical analysis: The analyses appear on the whole appropriate. However, the use of follow-up simple effects despite insignificant interaction effects for LOFC during REVDPDT was inappropriate. Unfortunately, this effect forms a cornerstone for the inference that LOFC broadly contributes to punishment discounting. That said, the LOFC REVDPDT simple effect is entirely consistent with the valid conclusions from LOFC DPDT, which authors invoke to justify the expedient LOFC REVDPDT follow-up tests. Perhaps more explicit acknowledgement of this in Discussion and more tempered language about LOFC contributions throughout manuscript would adequately address this issue.

The analyses appear to rely on extrapolation of missing datapoints. I assume this is done by imputing the missing data point from a linear curve fit to available datapoints. It is unclear whether this provides the best estimate of missing data, especially when considering discounting is not considered a linear function of time and the unclear relationship between 16s delay and no shock conditions. Another potential consequence of this procedure is that it may inflate the chance of detecting block effects by artificially promoting conformity in the data. A fuller characterization is needed to validate this approach.

There also appears to be some minor discrepancies in reported degrees of freedom for t-tests.

General Review:

The manuscript is well-written, the experiments are well-designed and the findings are interesting. As mentioned above, my main issues are that the analyses may not be appropriate, and that important aspects of the protocol remain unclear to me. Some comments/queries:

- Is the same lever associated with punishment across sessions and blocks? This should be made clear in Methods.

- What were the parameters for omissions? Could an animal have an omission during forced choice trials? These need to be detailed in Methods.

- Methods should describe how extrapolation was performed (linear slope?). Is there any data that shows this provides a good estimate for choice behaviour per delay condition, within-subjects, relative to other fitting procedures?

- Results should include how many animals were excluded on basis of omissions, and how many datapoints per delay condition were derived by extrapolation. Were complete omission blocks included in omission analyses and Figure 4?

- It is a bit unclear to me what is meant in lines 191-192. Does a preference within 0% and 100% mean each block were not at floor or ceiling per animal, i.e. every rat made at least one safe and one punished choice per block?

- Results should compare the final shock intensity per group. This is needed to support claims that sex differences were not observed due to shock titration (line 428-430).

- Degrees of freedom on some tests do not appear to correspond to the number of animals in those experiments. E.g. DPDT: N = 26, yet t(26) for training.

- The lack of BLA effect on choice of immediate punishment was highly surprising, given an increase in risky choice is typically observed under similar conditions, e.g. guaranteed immediate punishment in probabilistic decision-making task. The discussion around this was thin but arguably sufficient. I am curious whether there were any difference in effect across anterior-posterior axis of BLA, considering evidence for functional dissociations between aBLA vs pBLA in related tasks.

- Relatedly, Figure 2 should break down placements according to DPDT and REVDPDT. Figure 2 should also display mm from bregma to assist readers.

Minor comments/queries:

- line 64-65: is there any evidence that substance misuse is due to failures in consideration of subsequent detrimental outcomes?

- line 95-96: is delayed reward discounting a “critical component” of delayed punishment if these two processes are uncorrelated, as stated on line 84-85?

- line 125: BLA should be -2.8mm AP?

Reviewer #2

This study was designed to assess the role of the basolateral amygdala and the orbitofrontal cortex in how delay discounting of punishments interacts with appetitive-based decision making. Using a task recently developed by the group, the authors provide strong evidence implicating the orbitofrontal cortex, and to a lesser extent the basolateral amygdala, in the assessment of multi-featured offers that include a variably delayed punishment. To measure this, rats were presented with two options across two separate levers. One lever was statically reinforced with a small reward across the session, whereas the second lever was reinforced with a multi-featured outcome - an immediate large reward, and a mild shock, the temporal delay of which was systematically varied across the session. A muscimol/baclofen cocktail infused into the LOFC lowered rats’ choice for the large reward with punishment option, and critically, this effect was nearly identical regardless of whether the delayed punishment increased from a short to long delay or decreased from long to short, indicating this was not due to the nature of the serially presented delays in punishment. The authors also performed an identical pharmacological inactivation of the BLA, although a deficit was only observed during the sessions with increasing delays.

The manuscript is well-written and organized which made the experiment and results easy to follow and clear. The experiment was carefully designed, I commend the authors for including the REVDPDT design which provides a critical control experiment that permits a much clearer interpretation of the results. The titration of the shock magnitude for each rat was also a commendable decision by the authors, I imagine this contributed to the very nice range in the choice curves that the authors have observed. Overall I believe the manuscript is in fine condition, and I only have a few minor comments for the authors, mainly regarding the discussion.

1. In the discussion, the authors make a strong argument that LOFC inactivation disrupts delayed discounting of punishments, particularly in light of the symmetric results of inactivation of LOFC in the ascending and descending versions of the task. Although I think the authors have very solid ground upon which to base this argument, I believe that it’s important to note that this task does not just assess delay discounting of punishment on its own, but does so on a backdrop of reward-based decision making. This makes me wonder whether LOFC is critical for assessment of discounted delays of punishments specifically or whether it is required for integrating the disparate possible outcomes (the three pellets with the delayed shock), or put another way - assessing the overall expected value of a goods bundle to borrow from the economic decision-making literature. The observation that only substantially delayed punishments reveal a separation between LOFC inactivation and saline controls indicate that LOFC would not always be required for integrating the values. In parallel with the authors’ interpretations, it’s possible that LOFC is only required for integration of multiple features of an offer when delay discounting is required.

2. In the BLA REVDPDT inactivation experiment, the saline controls only drop to 50% choice of the shock + reward offer for the short delays. This is substantially higher than the LOFC saline controls in the same experiment. Is it possible that BLA inactivation lead to lower perceived aversiveness of the shock leading to greater choice of the reward + punishment offer? One might expect to see this in the ascending delay design as well, but in that experiment the rats have less experience with shock at the beginning of the session allowing for less opportunity for learning. Regardless of why the rats only drop to around 50% choice, this makes it a bit more difficult to interpret the BLA inactivation results on the REVDPDT experiment.

3. I was surprised that although there were similar patterns of BLA and LOFC inactivation on choice behavior on the DPDT task, the patterns of omissions across the two were substantially different. Do the authors have any ideas as to why this might be the case?

4. Could the authors provide A/P locations on the histology in figure 1? This would make it easier to get a sense of the final placements of the cannulae.

## Author Response

We thank the reviewers and editor for their positive tone and constructive feedback. Below is a point-by-point response to each comment (responses in bold italics). All changes and additions are highlighted in yellow in the manuscript.

Reviewer 1

1) Regarding statistical analysis: The analyses appear on the whole appropriate. However, the use of follow-up simple effects despite insignificant interaction effects for LOFC during REVDPDT was inappropriate. Unfortunately, this effect forms a cornerstone for the inference that LOFC broadly contributes to punishment discounting. That said, the LOFC REVDPDT simple effect is entirely consistent with the valid conclusions from LOFC DPDT, which authors invoke to justify the expedient LOFC REVDPDT follow-up tests. Perhaps more explicit acknowledgement of this in Discussion and more tempered language about LOFC contributions throughout manuscript would adequately address this issue.

We agree that the lack of significant LOFC x block interaction for REVDPDT diminishes the ability to draw strong interpretations from these data. We have added this caveat in the discussion, as well as the suggestion that decision-making with descending delays may be less sensitive to manipulations than ascending delays (see discussion p.19, 21, 25).

2) The analyses appear to rely on extrapolation of missing datapoints. I assume this is done by imputing the missing data point from a linear curve fit to available datapoints. It is unclear whether this provides the best estimate of missing data, especially when considering discounting is not considered a linear function of time and the unclear relationship between 16s delay and no shock conditions. Another potential consequence of this procedure is that it may inflate the chance of detecting block effects by artificially promoting conformity in the data. A fuller characterization is needed to validate this approach.

We thank the reviewer for bringing up this important point and are happy to elaborate. First and foremost: the amount of extrapolation in this manuscript was minimal, with only one block of missing data for LOFC REVDPDT and BLA DPDT, and no missing points of data extrapolated for LOFC DPDT and BLA REVDPDT. Notably, to avoid overuse of extrapolation, in instances when a subject omitted more than two blocks of trials, we ran that subject in another untreated baseline session, then repeated the treatment. We now clarify this on pp.10-11 of methods.

We agree that reward delay discounting often manifests as a hyperbolic rather than linear curve. However, in the two instances of fully omitted blocks of data included in this manuscript, we decided upon using linear slope to estimate that data point. This was based upon DPDT data from Liley et al. (2019), which utilized a progression of relatively short delays comparable to the current study (0,4,8,12,16, no shock). First, we noted that the mean slope was qualitatively linear (see Figure A below); then, we calculated the slope for each individual rat from multiple sets of blocks (blocks 1-6, 2-6, 3-6, 4-6, and 5-6). If data were indeed linear, one would expect that these slopes would be correlated (e.g., blocks 1-6 should be comparable to the slope from blocks 3-6). We ran these correlations and observed that the majority of these were highly significant (p<.01). The only exception was comparing blocks 1-6 with 5-6; this was non-significant due to ceiling effects, as slope flattens out once a subject hits ceiling (see Figure B below). However, our missing data strategy still effectively addresses this by filling in missing data with 100% if the previous point was also 100%. We now provide rationale for the choice of linear slope and acknowledge the paucity of omitted blocks on pp. 10-11 in Methods.

Figure modified from Liley, et al. (2019), eNeuro.

3) Is the same lever associated with punishment across sessions and blocks? This should be made clear in Methods.

Yes, lever identity is fixed throughout the experiment. We now clarify this in the methods on p.9.

4) What were the parameters for omissions? Could an animal have an omission during forced choice trials? These need to be detailed in Methods.

Trials were recorded as “omissions” in two possible scenarios: 1) rat failed to poke into the lit port to begin the trial during the allotted 10 seconds, or 2) rat poked to extend levers extend but did not choose a lever in 10 seconds. Omissions could also occur during forced choice trials; however, data depicted in figures 4, 6, 8, and 10 only include omissions of free choice trials. As mentioned above, if 20 or more trials in any pharmacology testing session were omitted, that session was repeated after another baseline session. This occurred for 1 rat in LOFC DPDT, 4 rats in LOFC REVDPDT, 1 rat in BLA DPDT, and 0 rats in BLA REVDPDT. This information has been added to Methods on pp.10-11.

5) Methods should describe how extrapolation was performed (linear slope?). Is there any data that shows this provides a good estimate for choice behaviour per delay condition, within-subjects, relative to other fitting procedures?

See detailed response to #2 above.

6) Results should include how many animals were excluded on basis of omissions, and how many datapoints per delay condition were derived by extrapolation. Were complete omission blocks included in omission analyses and Figure 4?

In the previous draft, we reported that subjects with > two blocks of omissions were excluded from the study, but this was inaccurate. Instead, these subjects were given another baseline session, then repeated the treatment session (See point 2 above). This occurred with five subjects across all experiments.

In the rare occurrence of a completely omitted block, this was scored as 10 omissions in that block during comparisons shown in Figures 4, 6, 8, 10. As stated above, there was only one block of missing data each for LOFC REVDPDT and BLA DPDT, and no fully omitted blocks for LOFC DPDT and BLA REVDPDT. This information has been added to the Methods (p.10-11).

7) It is a bit unclear to me what is meant in lines 191-192. Does a preference within 0% and 100% mean each block were not at floor or ceiling per animal, i.e. every rat made at least one safe and one punished choice per block?

We apologize for the lack of clarity. The criteria were not this rigid; it was acceptable for individual blocks to be at floor or ceiling. This is particularly difficult to avoid in the first and final blocks (which were often around 0%/100% in DPDT and 100%/0% in REVDPDT). Rather, mean choice across the entire session was required to be between floor (0% choice of punishment) and ceiling (100% choice of punishment). In addition, we also required that the session have a positive slope for DPDT and negative slope for REVDPDT. Between these two criteria, all subjects produced a robust discounting curve with sufficient parametric space for treatment to increase or decrease choice of delayed punishment. This has been updated in the methods on p.10.

8) Results should compare the final shock intensity per group. This is needed to support claims that sex differences were not observed due to shock titration (line 428-430).

Based on this suggestion, we performed a two-way analysis of variance to observe the impact of sex and task (DPDT vs REVDPDT) on differences in titration of foot shock mA (0.05 - 0.55). Surprisingly, there was not a significant difference between males and females, although there was a trend toward males having a higher terminal shock intensity (male DPDT: .28; male REVDPDT: .30; female DPDT: .24; female REVDPDT: .27; F(1, 53) = 2.757, p = .10). However, it is important to note that there were 17 more males than females overall, which likely contributed to the lack of main effect of sex. Furthermore, several females were unable to complete acquisition of the task, remaining at 0% choice of the large reward or omitting an excessive number of trials despite low shock intensity. Had these subjects been included, it is likely that we would have replicated the sex effect observed in Liley et al. (2019). This has been added to Results section on p.13 and is discussed on p. 23.

9) Degrees of freedom on some tests do not appear to correspond to the number of animals in those experiments. E.g. DPDT: N = 26, yet t(26) for training.

This discrepancy was present because some animals that completed task acquisition were unable to complete inactivation testing due to the Covid-19 laboratory shut down. We have removed these subjects from shaping analyses, and the statistical effects were comparable to the previous version (no differences between sexes to complete shaping procedures; significant sex difference in days taken to obtain stable behavior for DPDT; no sex difference in days to reach stability for REVDPDT). To maintain consistency with the inactivation experiments, we have removed the additional subjects from the results (pp.12-13).

10) The lack of BLA effect on choice of immediate punishment was highly surprising, given an increase in risky choice is typically observed under similar conditions, e.g. guaranteed immediate punishment in probabilistic decision-making task. The discussion around this was thin but arguably sufficient. I am curious whether there were any difference in effect across anterior-posterior axis of BLA, considering evidence for functional dissociations between aBLA vs pBLA in related tasks.

We agree that this lack of effect on immediate punishment choice across both tasks was surprising, and to determine if this lack of effect was related to BLA subregion, we classified cannula placements (total n = 29) as either anterior/rostral (>-2.6 from bregma, n = 4), posterior/caudal (<-2.6 from bregma, n = 10), or centralized (placement was apparent throughout both locations, n = 15). This division was based on Jean-Richard-Dit-Bressel & McNally, 2015, who determined that caudal but not rostral BLA is involved with sensitivity to footshock punishment. After performing a repeated measures ANOVA on rats that ran BLA DPDT (n = 10), REVDPDT (n = 10), or both tasks (n = 9), we found no significant effect of cannula placement (p = .550) or infusion x cannulae placement (p = .426). However, due to the high number of subjects with centralized placements, this dataset is not ideal for reliably comparing anterior vs posterior BLA function.

We now discuss the possibility of cannula placement causing the lack of BLA effects on sensitivity to immediate punishment, and compare our data to Jean-Richard-dit-Bressel and McNally (2015) and Orsini et al. (2015) in this context (Discussion p. 23-24).

11) Relatedly, Figure 2 should break down placements according to DPDT and REVDPDT. Figure 2 should also display mm from bregma to assist readers.

AP coordinates from bregma have been added to Figure 2 and placement colors have been updated to reflect rats that either ran in both tasks, only DPDT, or only REVDPDT.

12) line 64-65: is there any evidence that substance misuse is due to failures in consideration of subsequent detrimental outcomes?

We have altered our phrasing from “failure to consider” to “underestimate” and added references supporting this claim to p.4.

13) line 95-96: is delayed reward discounting a “critical component” of delayed punishment if these two processes are uncorrelated, as stated on line 84-85?

We agree that this was worded too strongly given the lack of correlation between choice of delayed rewards and punishments, and verbiage has been altered on p.5. That being said, given the lack of existing data on sensitivity to delayed punishment, we believe it is reasonable to speculate that brain regions that provide information about delays in other tasks are also involved with processing delays in DPDT, even if it is encoded by distinct neurobiological mechanisms.

14) line 125: BLA should be -2.8mm AP?

Surgical coordinates have been updated on p.7. Importantly, this led to detection of an error in our coordinates, as we had updated our AP coordinates for BLA to -3.0 from -2.8 to improve accuracy early in the experiment. This is now corrected in the revised manuscript.

Reviewer 2:

1) In the discussion, the authors make a strong argument that LOFC inactivation disrupts delayed discounting of punishments, particularly in light of the symmetric results of inactivation of LOFC in the ascending and descending versions of the task. Although I think the authors have very solid ground upon which to base this argument, I believe that it’s important to note that this task does not just assess delay discounting of punishment on its own, but does so on a backdrop of reward-based decision making. This makes me wonder whether LOFC is critical for assessment of discounted delays of punishments specifically or whether it is required for integrating the disparate possible outcomes (the three pellets with the delayed shock), or put another way - assessing the overall expected value of a goods bundle to borrow from the economic decision-making literature. The observation that only substantially delayed punishments reveal a separation between LOFC inactivation and saline controls indicate that LOFC would not always be required for integrating the values. In parallel with the authors’ interpretations, it’s possible that LOFC is only required for integration of multiple features of an offer when delay discounting is required.

We thank the reviewers for this thoughtful comment, and agree that it is difficult to disentangle the effects of LOFC on delay discounting per se vs. integrated value. We have added discussion of the role of OFC in outcome integration/mapping task space and discuss our data within this context. See pp. 21-22.

2) In the BLA REVDPDT inactivation experiment, the saline controls only drop to 50% choice of the shock + reward offer for the short delays. This is substantially higher than the LOFC saline controls in the same experiment. Is it possible that BLA inactivation lead to lower perceived aversiveness of the shock leading to greater choice of the reward + punishment offer? One might expect to see this in the ascending delay design as well, but in that experiment the rats have less experience with shock at the beginning of the session allowing for less opportunity for learning. Regardless of why the rats only drop to around 50% choice, this makes it a bit more difficult to interpret the BLA inactivation results on the REVDPDT experiment.

We agree that there is a qualitative difference between the saline controls for BLA and LOFC. As these experiments utilized two separate groups, individual differences in the shock level required to elicit a curve may have accounted for this baseline difference. Importantly, there was no difference between the saline session and baseline performance prior to treatment in the BLA experiment (p = .781), suggesting that the level of choice seen in the REVDPDT BLA saline condition existed at baseline prior to any manipulations. We now address this on p.24 of the discussion.

3) I was surprised that although there were similar patterns of BLA and LOFC inactivation on choice behavior on the DPDT task, the patterns of omissions across the two were substantially different. Do the authors have any ideas as to why this might be the case?

We agree that this was an interesting result, with BLA inactivation increasing omissions in later trials with delayed shock (Figure 6) and LOFC inactivation increasing omissions with immediate shock (Figure 4). It is possible that LOFC inactivation is increasing the salience of punishment (as seen in Orsini et al. (2015), reflected as increased disengagement in the presence of punishment rather than changes in choice. Notably, there were differences in sample characteristics between experiments, as the BLA experiment had more females than LOFC (5 vs. 3), and females were more likely than males to omit trials after inactivation of either region. Further experiments with larger samples of female subjects will enable deeper investigation of this phenomenon. We now discuss this on p.25.

4) Could the authors provide A/P locations on the histology in figure 1? This would make it easier to get a sense of the final placements of the cannulae.

This has been added to figure 2 - also see our comparison between anterior and posterior BLA in response to Reviewer 1 Point #10 above.
