# Supplementary material for: Lateral Orbitofrontal Cortex and Basolateral Amygdala Regulate Sensitivity to Delayed Punishment during Decision-Making
Source: eNeuro. 2022 Sep 6;9(5):ENEURO.0170-22.2022. doi: 10.1523/ENEURO.0170-22.2022 (PMC9463980; doi:10.1523/ENEURO.0170-22.2022)
Supplement: Extended data Figure 3-1 — Statistics summarizing effects of LOFC and BLA inactivation on decision-making with immediate vs. delayed punishment. Data for these analyses are visualized in figures 3,5,7, and 9. Download Figure 3-1, DOC file. [file enu-eN-NWR-0170-22-s02.doc]

**Extended Data Figure 3-1.**

| **Figure 3: LOFC inactivation during DPDT** | | | | | | | |
| --- | --- | --- | --- | --- | --- | --- | --- |
| 3-way mixed ANOVA | Block:  *F*_(2.793, 27.931)_ = 26.736,  *p* < .001 | Inactivation:  *F*_(1, 10)_ = 5.888, *p* = .036 | Sex:  *F*_(1,10)_ = .018,  *p* = .897 | Block x Sex:  *F*_(5, 50)_ = 1.087, *p* = .379 | Block x Inactivation:  *F*_(5, 50)_ = 3.261, *p* = .013 | Sex x Inactivation:  *F*_(1, 10)_ = 1.024, *p* = .335 | Sex  x Inactivation x Block:  *F*_(5, 50)_ = .663, *p* = .653 |
| **Figure 5: LOFC inactivation during REVDPDT** | | | | | | | |
| 3-way mixed ANOVA | Block:  *F*_(5, 60)_ = 21.468, *p* < .001 | Inactivation:  *F*_(1, 12)_ = 1.920, *p* = .191 | Sex:  *F*_(1, 12)_ = 3.119, *p* = .103 | Block x Sex:  *F*_(5, 60)_ = .781, *p* = .567 | Block x Inactivation:  *F*_(5, 60)_ = 1.128, *p* = .355 | Sex x Inactivation: *F*_(1, 12)_ = .002, *p* = .961 | Sex  x Inactivation x Block:  *F*_(5, 60)_ = .381, *p* = .860 |
| **Figure 7: BLA inactivation during DPDT** | | | | | | | |
| 3-way mixed ANOVA | Block:  *F*_(5, 60)_ = 16.312, *p* < .001 | Inactivation:  *F*_(1, 12)_ = 1.800, *p* = .205 | Sex:  *F*_(1, 12)_ = .231, *p* = .639 | Block x Sex:  *F*_(5, 60)_ = .1.479, *p* = .210 | Block x Inactivation:  *F*_(5, 60)_ = 3.102, *p* = .015 | Sex x Inactivation:  *F*_(1, 12)_ = .420, *p* = .529 | Sex  x Inactivation x Block:  *F*_(5, 60)_ = 1.093, *p* = .374 |
| **Figure 9: BLA inactivation during REVDPDT** | | | | | | | |
| 3-way mixed ANOVA | Block:  *F*_(5, 65)_ = 12.065, *p* < .001 | Inactivation:  *F*_(1, 13)_ = .444, *p* = .517 | Sex:  *F*_(1, 13)_ = 2.489, *p* = .139 | Block x Sex:  *F*_(5, 65)_ = 1.005, *p* = .422 | Block x Inactivation:  *F*_(5, 65)_ = .427, *p* = .828 | Sex x Inactivation:  *F*_(1, 13)_ = .015, *p* = .905 | Sex  x Inactivation x Block:  *F*_(5, 65)_ = .905, *p* = .483 |
