# Supplementary material for: Lateral Orbitofrontal Cortex and Basolateral Amygdala Regulate Sensitivity to Delayed Punishment during Decision-Making
Source: eNeuro. 2022 Sep 6;9(5):ENEURO.0170-22.2022. doi: 10.1523/ENEURO.0170-22.2022 (PMC9463980; doi:10.1523/ENEURO.0170-22.2022)
Supplement: Extended data Figure 4-1 — Statistics summarizing effects of LOFC and BLA inactivation on trial omissions. Data for these analyses are visualized in figures 4,6,8, and 10. Download Figure 4-1, DOC file. [file enu-eN-NWR-0170-22-s03.doc]

**Extended Data figure 4-1.**

| **Figure 4: LOFC inactivation omissions during DPDT** | | | | | | | |
| --- | --- | --- | --- | --- | --- | --- | --- |
| 3-way mixed ANOVA | Block:  *F*_(1.706, 17.061)_ = 5.734, *p* = .015 | Inactivation:  *F*_(1, 10)_ = 10.494, *p* = .009 | Sex:  *F*_(1, 10)_ = 4.395, *p* = .062 | Block x Sex:  *F*_(1.706, 17.061)_ = 3.516, *p* = .059 | Block x Inactivation:  *F*_(1.590, 15.901)_ = 2.220, *p* = .148 | Sex x Inactivation:  *F*_(1, 10)_ = 9.655, *p* = .011 | Sex  x Inactivation x Block:  *F*_(5, 50)_ = 5.220, *p* < .001 |
| **Figure 6: LOFC inactivation omissions during REVDPDT** | | | | | | | |
| 3-way mixed ANOVA | Block:  *F*_(2.363, 28.355)_ = 4.277, *p* = .019 | Inactivation:  *F*_(1, 12)_ = 4.690, *p* = .051 | Sex:  *F*_(1, 12)_ = 11.974, *p* = .005 | Block x Sex:  *F*_(2.363, 28.355)_ = 2.763, *p* = .072 | Block x Inactivation:  *F*_(2.760, 33.122)_ = 2.160, *p* = .116 | Sex x Inactivation: *F*_(1, 12)_ = 4.887, *p* = .047 | Sex  x Inactivation x Block:  *F*_(5, 60)_ = 2.028, *p* = .087 |
| **Figure 8: BLA inactivation omissions during DPDT** | | | | | | | |
| 3-way mixed ANOVA | Block:  *F*_(1.260, 15.118)_ = 8.647, *p* = .007 | Inactivation:  *F*_(1, 12)_ = 3.470, *p* = .087 | Sex:  *F*_(1, 12)_ = 9.555, *p* = .009 | Block x Sex:  *F*_(1.260, 15.118)_ = 6.326, *p* = .018 | Block x Inactivation:  *F*_(2.356, 28.276)_ = 3.095, *p* = .053 | Sex x Inactivation:  *F*_(1, 12)_ = .502, *p* = .492 | Sex  x Inactivation x Block:  *F*_(5, 60)_ = 1.891, *p* = .109 |
| **Figure 10: BLA inactivation omissions during REVDPDT** | | | | | | | |
| 3-way mixed ANOVA | Block:  *F*_(2.405, 31.263)_ = 3.943, *p* = .023 | Inactivation:  *F*_(1.000, 13.000)_ = .070, *p* = .795 | Sex:  *F*_(1, 13)_ = 1.660, *p* = .220 | Block x Sex:  *F*_(2.405, 31.263)_ = 1.316, *p* = .285 | Block x Inactivation:  *F*_(2.199, 28.589)_ = 1.082, *p* = .357 | Sex x Inactivation:  *F*_(1, 13)_ = .695, *p* = .419 | Sex  x Inactivation x Block:  *F*_(5, 65)_ = .195, *p* = .963 |
