# Supplementary material for: Lateral Orbitofrontal Cortex and Basolateral Amygdala Regulate Sensitivity to Delayed Punishment during Decision-Making
Source: eNeuro. 2022 Sep 6;9(5):ENEURO.0170-22.2022. doi: 10.1523/ENEURO.0170-22.2022 (PMC9463980; doi:10.1523/ENEURO.0170-22.2022)
Supplement: Extended data Figure 4-2 — Statistics summarizing effects of LOFC and BLA inactivation on choice latency. Data for these analyses are visualized in figures 4,6,8, and 10. Download Figure 4-2, DOC file. [file enu-eN-NWR-0170-22-s04.doc]

**Extended Data figure 4-2**

| **Figure 4: LOFC inactivation latency during DPDT** | | | | | | | |
| --- | --- | --- | --- | --- | --- | --- | --- |
| 3-way mixed ANOVA | Block:  *F*_(1, 11)_ = .073, *p* = .792 | Inactivation:  *F*_(1, 11)_ = .003, *p* = .960 | Sex:  *F*_(1, 11)_ = 8.871, *p* = .013 | Block x Sex:  *F*_(1, 11)_ = 2.319, *p* = .156 | Block x Inactivation:  *F*_(1, 11)_ = 4.435, *p* = .059 | Sex x Inactivation:  *F*_(1, 11)_ = 2.368, *p* = .152 | Sex  x Inactivation x Block:  *F*_(1, 11)_ = .695, *p* = .422 |
| **Figure 6: LOFC inactivation latency during REVDPDT** | | | | | | | |
| 3-way mixed ANOVA | Block:  *F*_(1, 11)_ = 11.619, *p* = .006 | Inactivation:  *F*_(1, 11)_ = 3.403, *p* = .092 | Sex:  *F*_(1, 11)_ = 11.420, *p* = .006 | Block x Sex:  *F*_(1,11)_ = 1.911, *p* = .194 | Block x Inactivation:  *F*_(1, 11)_ = .300, *p* = .595 | Sex x Inactivation:  *F*_(1,11)_ = 2.745, *p* = .126 | Sex  x Inactivation x Block:  *F*_(1, 11)_ = .003, *p* = .956 |
| **Figure 8: BLA inactivation latency during DPDT** | | | | | | | |
| 3-way mixed ANOVA | Block:  *F*_(1,12)_ = .770, *p* = .397 | Inactivation:  *F*_(1, 12)_ = 1.339, *p* = .270 | Sex:  *F*_(1, 12)_ = 12.551, *p* = .004 | Block x Sex:  *F*_(1,12)_ = .223, *p* = .645 | Block x Inactivation:  *F*_(1, 12)_ = .178, *p* = .680 | Sex x Inactivation:  *F*_(1,12)_ = .016, *p* = .903 | Sex  x Inactivation x Block:  *F*_(1, 12)_ = .391, *p* = .544 |
| **Figure 10: BLA inactivation latency during REVDPDT** | | | | | | | |
| 3-way mixed ANOVA | Block:  *F*_(1, 11)_ = .073, *p* = .792 | Inactivation:  *F*_(1, 11)_ = .003, *p* = .960 | Sex:  *F*_(1, 11)_ = 8.871, *p* = .013 | Block x Sex:  *F*_(1, 11)_ = 2.319, *p* = .156 | Block x Inactivation:  *F*_(1, 11)_ = 4.435, *p* = .059 | Sex x Inactivation:  *F*_(1,11)_ = 2.368, *p* = .152 | Sex  x Inactivation x Block:  *F*_(1, 11)_ = .695, *p* = .422 |
